# Effect of goal-directed mobilisation versus standard care on physical functioning among medical inpatients: the GoMob-in randomised, controlled trial

**DOI:** 10.1136/bmjopen-2024-086921

**Published:** 2024-11-14

**Authors:** Fabian D Liechti, Jeannelle Heinzmann, Nina A Schmutz, Michael L Rossen, Jean-Benoît Rossel, Andreas Limacher, Joachim M Schmidt Leuenberger, Christine Baumgartner, Maria M Wertli, Drahomir Aujesky, Martin Verra, Carole E Aubert

**Affiliations:** 1 Department of General Internal Medicine, Inselspital, Bern University Hospital, University of Bern, Bern, Switzerland; 2 CTU Bern, Department of Clinical Research, University of Bern, Bern, Switzerland; 3 Department of Physiotherapy, Inselspital, Bern University Hospital, University of Bern, Bern, Switzerland; 4 Department of General Internal Medicine, Kantonsspital Baden, Baden, Switzerland; 5 Institute for Primary Healthcare, University of Bern, Bern, Switzerland

**Keywords:** Hospitalization, Physical Therapy Modalities, Quality in health care, Aged, Hospital to Home Transition

## Abstract

**Objective:**

To assess the effect of goal-directed mobilisation (GDM) on physical functioning in medical inpatients.

**Design:**

Randomised, controlled, single-centre, parallel, superiority trial with a 3-month follow-up and blinded outcome assessment.

**Setting:**

General internal medicine wards of a Swiss tertiary acute hospital, September 2021 to April 2023.

**Participants:**

Adults with expected hospitalisation of ≥5 days, physiotherapy prescription and ability to follow study procedures.

**Intervention:**

GDM during hospitalisation, which includes personal goal setting and a short session of patient education through a physiotherapist (experimental group), versus standard care (control group).

**Outcome measures:**

The primary outcome was the change in physical activity between baseline and day 5 (De Morton Mobility Index (DEMMI)). Secondary outcomes included in-hospital accelerometer-measured mobilisation time; in-hospital falls; delirium; length of stay; change in independence in activities of daily living, concerns of falling and quality of life; falls, readmission and mortality within 3 months.

**Results:**

The study was completed by 123 of 162 (76%) patients enrolled, with the primary outcome collected at day 5 in 126 (78%) participants. DEMMI Score improved by 8.2 (SD 15.1) points in the control group and 9.4 (SD 14.2) in the intervention group, with a mean difference of 0.3 (adjusted for the stratification factors age and initial DEMMI Score, 95% CI −4.1 to 4.8, p=0.88). We did not observe a statistically significant difference in effects of the interventions on any secondary outcome.

**Conclusions:**

The patient’s physical functioning improved during hospitalisation, but the improvement was similar for GDM and standard of care. Improving physical activity during an acute medical hospitalisation remains challenging. Future interventions should target additional barriers that can be implemented without augmenting resources.

**Trial registration number:**

NCT04760392.

**STRENGTHS AND LIMITATIONS OF THIS STUDY**

- We recruited from general internal medicine wards and used broad inclusion criteria to investigate the intervention in a diverse population.
- The investigation did not use additional resources.
- Participants were randomised, the primary outcome assessment was blinded and models were adjusted for stratification factors and baseline values.
- Key limitation is the high number of participants with a hospitalisation shorter than expected and, hence, missing primary outcome assessment.
- Further, due to randomisation on an individual level, our intervention may also have influenced treatment in the standard care group despite high adherence to the intervention.

## Introduction

 Low physical activity during hospitalisation is associated with cascading adverse effects, such as falls, depression, institutionalisations, readmissions and mortality.^1^,^4^ While bedrest is rarely indicated, medical inpatients still spend a lot of time in bed, with older patients spending up to 80% of the time there.^5 6^ The low level of mobility during hospitalisation can be explained by multiple barriers at patient, healthcare professional, environment and system levels and the low priority of patient mobilisation.^7 8^

Interventions aimed at improving mobility during hospitalisation can lead to better outcomes, such as decreased loss of autonomy, falls, costs and length of stay and fewer readmissions and institutionalisations.^9^,^20^ Among those interventions, goal-directed mobilisation (GDM) programmes, where a mobility goal is defined and communicated to the patients and their treating team, were associated with increased physical activity, but evidence on its benefit remains limited.^19 20^ GDM is usually started early during hospitalisation or intensive care treatment, where it has been shown to improve physical activity in selected patient groups.^21 22^ A recent review summarised limitations that could explain why broad-scale practice changes and implementation of GDM did not occur: studies aimed at increasing mobility during hospitalisation often required additional staff, follow-up was frequently limited to hospitalisation duration and the studies presented methodological limitations, such as a pre–post design or a small sample size.^21^ Also, the effects of GDM in general internal medicine inpatients—a large, quickly growing, and resource-intensive inpatient group, many of whom suffer from chronic diseases and fatigue and are multimorbid and highly complex—is uncertain.^23^,^25^

To address previous studies’ limitations and the evidence gap regarding GDM in a diverse patient population, we conducted the *go*al-directed *mobilisation* versus standard care on physical functioning among medical *in*patients trial, which tested the effect of a GDM intervention compared with standard care on physical functioning during hospitalisation and other outcomes within 3 months. The GDM programme was developed to not require additional resources.

## Materials and methods

### Study design

We performed a randomised, controlled, single-centre, open-label, parallel, superiority trial with a 3-month follow-up and a blinded outcome assessment, registered before start of recruitment at ClinicalTrials.gov (NCT04760392) and conducted according to the published protocol.^26^ Reporting follows the Consolidated Standards of Reporting Trials 2010 statement recommendations.^27^ During the consent process, all patients received information on the general aims of the study, randomisation and planned assessments, but not on the specific instructions used in the intervention.

### Patient and public involvement

None.

### Participants

We enrolled patients aged ≥18 years, who were hospitalised in the department of general internal medicine, Bern University Hospital (tertiary acute care hospital in Switzerland) and had a physiotherapy prescription. Exclusion criteria were (1) inability to follow study procedures or sign informed consent due to language problems (unable to understand German), psychological disorders, severe dementia^28^ or blindness; (2) expected hospital stay<5 days (based on clinical evaluation by the study staff and discussion with a senior physician); (3) medically indicated bedrest or restrictions due to contact precautions for >24 hours; (4) lower extremity deficits directly impairing walking capacity, for example, after stroke or fracture; (5) terminal illness; (6) pregnancy/breastfeeding. Compared with the original protocol, we dropped the criterion of only including one patient per room after March 2022 to increase recruitment because patients were often moved to other rooms and staff was not attributed to the same rooms each following day. Baseline characteristics (demographic data, prehospital mobility, De Morton Mobility Index (DEMMI) Score, Barthel Index, Falls Efficacy Scale—International (FES-I) and the 5-level version of the EuroQol EQ-5D questionnaire (EQ-5D-5L)) were collected before randomisation. The DEMMI is a 15-item score to measure physical functioning in older hospitalised adults (0, poor physical functioning to 100, independent activity) and has a minimal clinically important difference of 5–10 points.^29^,^31^

### Randomisation and blinding

Patients were randomly assigned (1:1) to the experimental or control group, at latest on the second hospitalisation day using the data entry software REDCap (Vanderbilt University, Nashville, Tennessee, USA)^32^ with stratification by baseline DEMMI Score (≤40 vs >40 points; online supplemental materials) and age (<65 vs ≥65 years old) using varying block sizes of 2, 4 and 6.^29 30^ The allocation sequence (randomisation list) was concealed from the study team. All participants and the study team, except physiotherapists assessing the DEMMI and statisticians responsible for the final analysis, were aware of group assignment after baseline assessment and randomisation.

### Intervention

The experimental intervention consisted of a GDM programme during hospitalisation. After randomisation, the study team assigned in discussion with the patient the first individual mobility goal. Mobility goals were adapted from the Johns Hopkins Highest Level of Mobility and used eight levels (1, bed activities; 2, sit at edge of bed; 3, transfer to chair/commode; 4, stand for >1 min; 5, walk≥10 steps; 6, walk≥7.5 m; 7, walk≥75 m; 8, walk≥75 m (30 min or stairs) and no bed rest during daytime).^33^ Participants were instructed to complete the mobility goal at least three times per day. The identified individual mobility goal was then depicted on a patient communication board next to the bed, visible for all stakeholders (patients, visiting friends and family, physicians, nurses, physiotherapists; online supplemental table 1). Participants in the intervention group received a leaflet with a guide on importance, aims and implementation of early mobilisation during hospitalisation, including definitions of mobility goals (online supplemental materials). During the first physiotherapy session (at latest on the first working day after study inclusion), a trained physiotherapist provided short education on GDM. The physiotherapist revised the individual mobility goal level, in discussion with the patient and taking into account current mobility capacities, comorbidities and perspectives of recovery. The frequency and total number of physiotherapy sessions was at the discretion of the treating physiotherapists. The number of physiotherapy sessions was retrieved from electronic health records at the end of the study. To avoid using additional resources, that is, increasing the amount of physiotherapy provided, the intervention focussed on indicating a clear goal to increase mobilisation throughout the hospital stay, particularly between physiotherapy sessions, when no specific staff was available. The intervention was formally stopped at hospital discharge, transfer to a unit other than general internal medicine or 15 days after study inclusion. Patient communication boards were removed, and goal setting not anymore actively encouraged, but, if necessary, patients would still receive routine physiotherapy. All healthcare professionals caring for the participant were allowed to adapt the mobility goal level together with the patient at any time—usually to a higher level, but level reductions were also allowed.

The study team instructed nurses and physicians about the trial’s aims without using resources not available in everyday practice: before inclusion of the first participant, nurses were instructed by brief oral presentations at their regular meetings and thereafter every 3–4 months and through disseminated handouts. Residents received a brief email at inclusion of a patient to the intervention group. Trained physiotherapists with experience in general internal medicine were instructed at trial inception and thereafter if deemed necessary (eg, for new staff members or after protocol deviations). Protocol adherence was assessed at study visits by presence of the board displaying the mobility goal level and by nurses’ and physiotherapists’ entries to the electronic health record to detect any protocol deviations.

### Control intervention and standard care

All patients in both groups received standard care, including physiotherapy. Regular physiotherapy sessions included individual training and/or group sessions, while the content and frequency were determined by the physiotherapists after initial prescription by a physician according to the patient’s abilities and individual goals.

### Contrasts between interventions

Mobility goals were not routinely set nor encouraged interprofessionally in the control group and not displayed on the patient communication board. The medical team was not informed about patients recruited to the control group.

### Outcome measures

The primary outcome was the change in physical functioning between baseline and day 5±2 days (D5) after enrolment, measured with the DEMMI by trained study staff blinded to group allocation. Shortly before the assessment, patients were instructed not to reveal their group allocation; patient communication boards indicating mobility goals were then removed by nursing staff for the time of the assessment by the physiotherapist to minimise the risk of revealing group allocation to the assessor.

All participants were equipped with a waterproof, wrist-worn accelerometer (GENEActiv) to measure three axes of acceleration at a frequency of 50 Hz, allowing uninterrupted data collection for 15 days, along with temperature to verify that the device was worn continuously.^34^ The accelerometer has no display because the study’s aim was to evaluate implementation of GDM without using additional technological assets, which has already been proven to function, but comes at additional costs.^9 35^ The accelerometers were activated when participants were fitted with them and collected by the study team when discharged from the hospital, transferred to a unit other than general internal medicine for longer than 24 hours, or 15 days after study inclusion (end of the intervention) to read out the data. Data were not analysed until they had been fully collected from all participants.

Secondary outcomes collected during the hospital stay or from discharge letters included change in DEMMI between baseline and discharge or day 14±2 days (D14; whichever occurred first, blinded assessment); accelerometer-measured mobilisation time between enrolment and D5, and between enrolment and discharge (blinded assessment); in-hospital falls; delirium; length of stay; and discharge destination (ie, home, rehabilitation clinic, nursing home or palliative care unit or hospital). Secondary outcomes collected by phone interview 3 months after randomisation included the number of falls and readmissions; all-cause mortality; and change between enrolment and 3-month follow-up in Barthel Index (0–100 points, higher scores indicate greater independence in activities of daily living),^36^ concerns of falling (FES-I, 16–64 points, higher scores indicating higher concern)^37^ and quality of life (EQ-5D-5L, mobility dimension (levels 0–5) and Visual Analogue Scale (score 0–100, higher scores indicating better quality of life)).^38^ Mobilisation time was calculated from accelerometer data by a statistician blinded to the group allocation and defined as proportion of time with light, moderate or vigorous physical activity (acceleration>30 mG), disregarding nocturnal sleeping time and the time not wearing the device (R statistical software, GGIR V.2.9.0).^39^

### Statistical analysis

We estimated that a sample size of 64 in each group (total 128 participants) would have a power of 80% to detect a difference in DEMMI change within 5 days of 5 score points, the minimal clinically important difference, with a SD of 10 points at a two-sided alpha level of 0.05.^29^,^3140 41^ To account for a dropout of 20%, we increased the sample size to 80 per group (total 160 participants).

Data analysis was conducted according to a prespecified statistical analysis plan (online supplemental materials) by a statistician without access to the group allocation until the primary analysis was finished. The primary intention-to-treat analysis used a linear model to assess changes in DEMMI with adjustments for stratification factors. Secondary continuous outcomes were analysed using the same approach. Quality of life was analysed as the difference between baseline and 3 months in the Visual Analogue Scale and in the mobility dimension of the EQ-5D-5L. Count outcomes were assessed using a negative binomial model, categorical outcomes using a multinomial logistic model, binary outcomes using logistic regression and time-to-event outcomes using a Cox regression model. All analyses were adjusted for the stratification factors; changes from baseline were additionally adjusted for the baseline value.

If outcomes were missing, we employed multiple imputation in the primary analysis, including the subgroup analyses, except for categorical and count outcomes, where only age, sex and body mass index were used in the imputation model. We used multiple imputation by chained equation to impute all variables separately, generating 50 imputed data set as predefined in the statistical analysis plan (online supplemental materials). In a secondary per-protocol analysis, without using imputation for missing values, patients who violated any eligibility criteria, did not receive the allocated intervention or were discharged before day 4 were disregarded.

We performed prespecified subgroup analyses for baseline DEMMI (≤40 vs >40), age (<65 vs ≥65 years) and prehospital mobility (no mobility aid vs use of mobility aid), because these variables are associated with lower DEMMI scores.^42 43^ All effect measures were accompanied by 95% CI. The statistical testing was two sided with a type 1 error of 5%. Analyses were performed with Stata V.18.0 and R V.4.2.2.

## Results

### Study population

**Figure 1:**
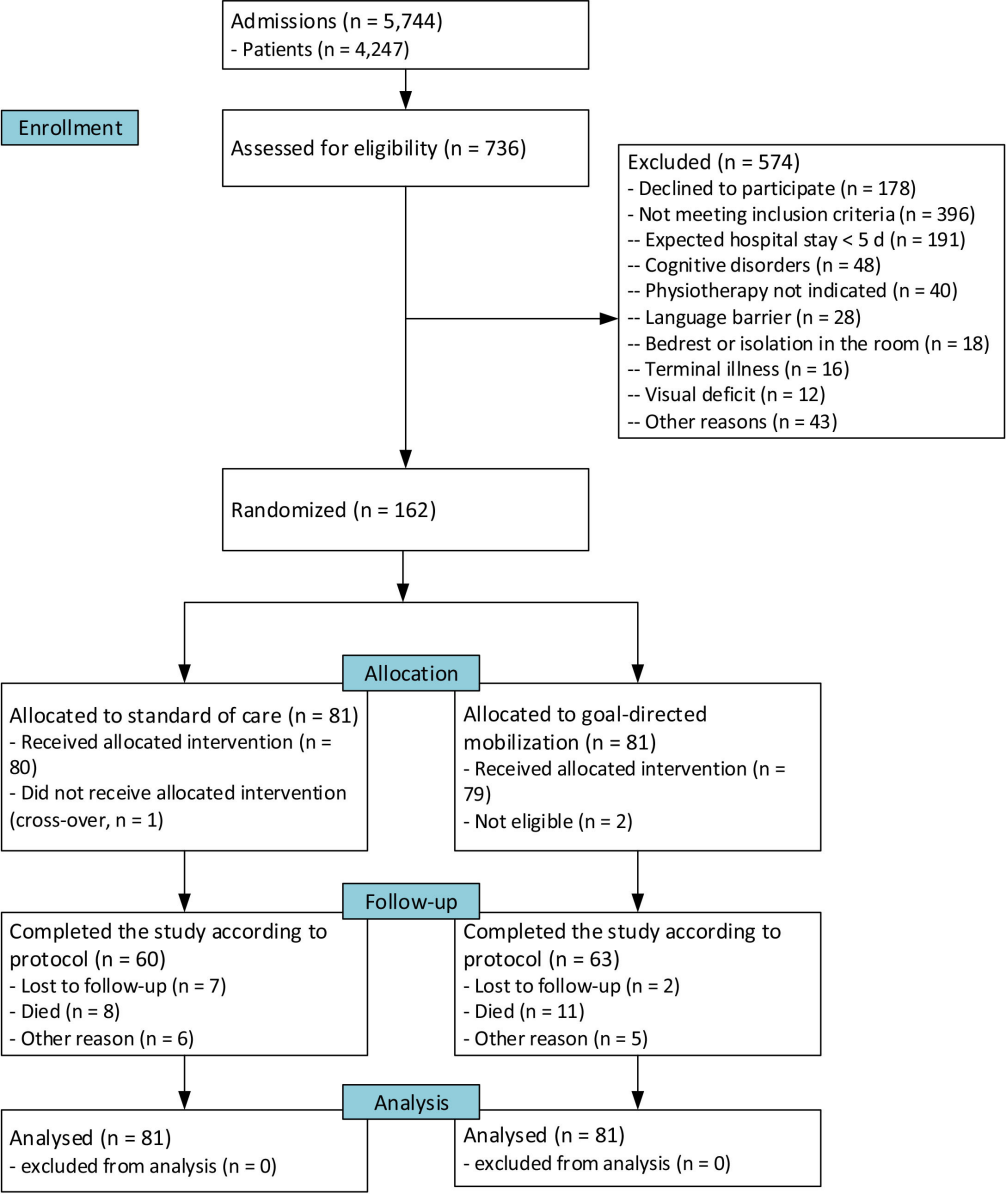
Flow of participants in the goal-directed mobilisation of medical inpatients trial (according to Consolidated Standards of Reporting Trials).

Between 12 September 2021 and 18 January 2023, we enrolled 162 patients among 5744 admissions to our department; 81 were randomised to receive standard care and 81 GDM (flow chart in figure 1). The median age was 72 (IQR 17, range 23–100) years; 97 (60%) participants were men and 73 (45%) used a walking aid before admission. Intervention and control groups were similar at baseline, except for older age and more self-reported depression in the intervention group, but not according to the depression diagnoses reported in the discharge letters (table 1 and online supplemental tables 2 and 3).

**Table 1:** Baseline characteristics of study participants by allocation group

Measures	Standard care (n=81)	Goal-directed mobilisation (n=81)
Age		
Mean (SD) (years)	70.0 (12.0)	72.6 (14.4)
Median (IQR) (years)	70 (64–79)	76 (63–82)
≥65 years old	58 (71.6%)	58 (71.6%)
Sex (male)	52 (64.2%)	45 (55.6%)
Body mass index, median (IQR) (kg/m^2^)	25.5 (22.5–30.8)	24.8 (21.5–29.8)
Elective admission	7 (8.6%)	5 (6.2%)
Intensive care before enrolment	5 (6.2%)	10 (12.3%)
Mobility aid		
None	52 (64.2%)	37 (45.7%)
Walking stick	12 (14.8%)	19 (23.5%)
Walking frame	17 (21.0%)	25 (30.9%)
Fall(s) during last 6 months		
None	53 (65.4%)	47 (58.0%)
1–2	16 (19.8%)	22 (27.2%)
>2	12 (14.8%)	12 (14.8%)
De Morton Mobility Index Score (range 0–100 points)		
Mean (SD)	58.8 (23.4)	55.5 (22.9)
Median (IQR)	62 (41–74)	57 (41–74)
≤40 points	19 (23.5%)	19 (23.5%)
Falls Efficacy Scale (range 16–64 points), median (IQR)	22 (16–31)	22 (17–30)
EQ-5D-5L		
Mobility dimension (range 0–5), median (IQR)	2 (1–3)	3 (1–4)
Visual Analogue Scale (range 0–100), median (IQR)	50 (39–68)	50 (40–70)
Barthel Index (range 0–100), median (IQR)	100 (90–100)	95 (80–100)

Data are median with IQR, mean with SD, or number with percentage.

EQ-5D-5L, 5-level version of the EuroQol EQ-5D questionnaire

### Procedural characteristics

Participants received the intervention or control procedure largely as intended, except in the intervention group where two patients interrupted participation due to new conditions prohibiting mobilisation and in the control group where one patient accidentally received instructions that included goal setting. We did not encounter any relevant serious adverse events. The first personal mobility goals are reported in table 2. The primary outcome was available in 137 patients. In 126 (78%) participants (control group, 61 participants (75%); intervention group 65 participants (80%)), DEMMI was assessed for the second time on D5. 19 (12%) participants died within the 3-month follow-up and 20 (12%) were lost to follow-up, leaving 123 participants completing the study according to protocol (60 participants in the control, 63 in the intervention group). This included three protocol deviations with one control group patient who accidentally received the intervention, and two intervention group participants who were not eligible because they participated in the study previously in the control group.

**Table 2:** First personal mobility goals of participants in the intervention group (baseline visit)

Level	Mobility goal	N (%)
1	Bed activities	8 (9.9%)
2	Sit at edge of bed	7 (8.6%)
3	Transfer to chair/commode	2 (2.5%)
4	Stand for >1 min	6 (7.4%)
5	Walk≥10 steps	11 (13.6%)
6	Walk≥7.5 m	18 (22.2%)
7	Walk≥75 m	29 (35.8%)
8	Walk≥75 m (30 min or stairs)	0 (0%)

The estimated discharge date was difficult to predict and the length of stay was shorter than expected in 27 participants (less than 3 days in 17 (21%) control group participants and in 10 (12%) intervention group participants). In 25% of the participants in the control group and 20% in the intervention group, the primary outcome was assessed outside the intended period (protocol deviations are listed in online supplemental table 4). Mobilisation time was recorded for at least 1 day in 141 (87%) participants. Reasons for failure to record included technical issues and participant’s refusal to wear the device. The number of physiotherapy sessions did not differ between groups (in both groups, median 3, IQR 2–5).

### Primary outcome

In the full-analysis set with multiple imputation, DEMMI Score improved by 8.2 (SD 15.1) points in the control and 9.4 (SD 14.2) points in the intervention group, without reaching statistical difference between the two groups (adjusted difference using multiple imputation 0.3, 95% CI −4.1 to 4.8, p=0.88; table 3). The participant’s individual DEMMI Score tracks varied considerably, but the proportion of participants with any DEMMI Score improvements was similar between groups (control group, 69%, intervention group 64%, adjusted OR 0.6, 95% CI 0.2 to 1.4, p=0.19; Online supplemental figures 1 and 2). In the full-analysis set of 137 available cases, including those with primary outcome assessment outside the intended time frame of D5, adjusted difference was 0.2 (95% CI −4.3 to 4.8, p=0.92).

**Table 3:** Primary and secondary outcome measures are shown as unadjusted values with missing data imputed (means with SD, proportions or median survival times)

Measures	Standard of care (n=81)	Goal-directed mobilisation (n=81)	Adjusted effect estimate (95% CI)*	P value
Change in De Morton Mobility Index (DEMMI) Score				
Baseline to day 5 (MD)	8.2 (15.1)	9.4 (14.2)	0.3 (−4.1 to 4.8)	0.88
Baseline to discharge (MD)	9.0 (15.9)	9.9 (14.4)	−0.1 (−4.7 to 4.5)	0.97
Improvement (baseline to day 5) (OR)	69.3%	63.7%	0.6 (0.2 to 1.4)	0.19
Mobilisation time, daily proportion of time spent active, measured by accelerometer (%)				
Until day 5 (MD)	13.4 (7.6)	14.9 (9.1)	1.5 (−1.1 to 4.2)	0.26
Until discharge (MD)	13.4 (7.6)	15.0 (9.2)	1.6 (−1.0 to 4.3)	0.23
Number of in-hospital falls (IRR)	0.0 (0.2)	0.0 (0.2)	1.0 (0.1 to 7.1)	1.00
In-hospital delirium (OR)	4.9%	6.2%	1.3 (0.3 to 5.0)	0.73
Length of stay, median (IQR) (HR)	5 (4, 9)	5 (4, 8)	1.0 (0.8 to 1.4)	0.86
Discharge destination (RR)			1.4 (0.7 to 2.7)	0.38
Home	60.0%	61.7%	1	Ref.
Rehabilitation clinic	15.3%	13.4%	0.8 (0.3 to 2.3)	0.75
Nursing home or palliative care	10.4%	8.1%	0.8 (0.2 to 2.5)	0.64
Other hospitals	14.3%	16.9%	1.1 (0.4 to 3.1)	0.80
Outcomes within 3 months				
Number of falls (IRR)	0.3 (0.8)	0.7 (2.4)	1.8 (0.8 to 3.9)	0.13
Number of readmissions (IRR)	0.7 (1.0)	0.7 (1.2)	0.9 (0.6 to 1.5)	0.79
All-cause mortality, cumulative incidence (95% CI) (HR)	10.6% (5.4% to 20.1%)	11.1% (5.9% to 20.3%)	1.1 (0.4 to 2.9)	0.80
Change from baseline to telephone interview†				
Barthel Index (range 100–0) (MD)	1.0 (18.5)	3.9 (23.9)	−0.3 (−6.0 to 5.5)	0.93
Falls Efficacy Scale (range 16–64 points) (MD)	−1.4 (9.6)	−2.9 (10.5)	−0.8 (−3.5 to 1.8)	0.54
EQ-5D-5L, Mobility Dimension (range 1–5) (MD)	−0.4 (1.5)	−0.5 (1.3)	0.0 (−0.4 to 0.4)	0.93
EQ-5D, Visual Analogue Scale (range 0–100) (MD)	9.6 (24.6)	7.2 (23.7)	−0.9 (−7.9 to 6.1)	0.81
Improvement in Barthel score (OR)	30.1%	37.5%	1.1 (0.5 to 2.6)	0.89

EQ-5D-5L, 5-level version of the EuroQol EQ-5D questionnaire

* Mean differences (MD), incidence- rate ratios (IRR), relative risk ratios (RR), OR, HR as appropriate; adjusted for baseline DEMMI sScore (≤40 points vs >40 points and age (<65 vs ≥65 years), changes from baseline additionally adjusted for the baseline value; .

† aAt 3 months.

### Secondary outcomes

No differences in DEMMI scores between study inclusion and discharge were observed. Until both, day 5 and discharge, the mobilisation time measured by accelerometers was similar in the control group and in the intervention group. Length of hospital stay was not influenced by the intervention and the proportion of patients discharged at home was similar in both groups. During the hospital stay, two falls were reported in each of the groups, both without severe complications.

Three months after study inclusion, we did not observe any differences in effect between the intervention and the control group, that is, adjusted differences for change in concerns of falling as measured by the FES-I, the EQ-5D-5L mobility dimension and Visual Analogue Scale did not change. 3-month readmission and mortality were similar in both groups. Readmission or death within 3 months was observed in 42 of 81 (52%) participants in the intervention group (11 deaths, 32 readmissions) and in 38 of 81 (47%) participants in the control group (eight deaths, 34 readmissions).

### Secondary analyses

In the per-protocol analysis, the primary analysis’ results of the primary outcome were confirmed (adjusted difference 0.6, 95% CI −4.5 to 5.8, p=0.81). In subgroup analyses, which were prescheduled in the published study protocol but underpowered, we observed a larger difference in participants younger than 65 years old (difference in DEMMI Score 5.0, 95% CI −4.5 to 14.5, p=0.29) compared with those at least 65 years old (−1.5, 95% CI −6.4 to 3.4, p=0.55); we also observed a larger difference in participants not using a mobility aid before admission (4.0, 95% CI −2.5 to 10.4, p=0.22) compared with those needing a mobility aid (−3.1, 95% CI −9.2 to 3.1, p=0.32), but the differences between the control and the intervention groups were not significant (online supplemental table 5).

## Discussion

In this randomised, controlled trial with blinded outcome assessment, we found no difference between GDM and standard of care on physical functioning levels measured by DEMMI during an acute care hospitalisation. Secondary outcomes—such as mobilisation time, length of hospital stay, discharge destination or 3-month survival—were not significantly influenced by the GDM programme.

Analyses in predefined subgroups revealed possible beneficial effects of GDM in younger participants and those without prior mobility aid; however, these analyses were underpowered and statistically non-significant. The finding that younger patients may profit more from GDM may be explained by the increasing proportion of cognitive impairment usually observed in older people, while patients’ prehospital use of mobility aids possibly indicates long-term mobility deficits.^44^ People with such impairments might need additional resources to improve physical activity or functioning. However, it is possible that they benefited from GDM, but recovery was too slow to be recorded.

Our negative findings contrast with results of small randomised trials.^13 15^ However, studies in medical wards using goal setting suffered methodological issues and failed to demonstrate positive long-term effects, and the only beneficial short-term effect consistently reported across studies was an increase in physical activity.^21^ Additionally, publication bias may play a role, as the number of studies with a randomised design in this field is generally low and funnel plot asymmetry is typical.^45^ Several factors may explain why GDM did not increase physical functioning.

First, the intervention might work for a specific subgroup of patients only, for example, younger patients or those with less comorbidities or complexity, as shown in the subgroup analysis. Individual trajectories of our participants indeed varied considerably. In such a heterogeneous population recruited with various ages and levels of morbidity, multiple barriers and facilitators should be addressed together—but this often requires more resources.

Second, the time window may have been too short for an effective intervention, for example, in a previous study, physical activity increased only after the third day of a GDM intervention, while a meta-analysis modelled increasing effectiveness after admission until 2 weeks after discharge.^17 46^ With the length of hospital stay becoming shorter, the time being physically active (measured as proportion of hospitalisation time) may remain low, despite improving physical activity.^6^ This can be explained by both the patients’ enfeeblement due to their presenting complaints and time-consuming investigations and treatments mostly during the beginning of hospitalisation. Nevertheless, 40% of our participants were transferred to another institution (eg, hospital, rehabilitation centre or a nursing home) after their index hospitalisation, where a goal-setting mobilisation could be continued to improve physical activity.

Third, our intervention may not have been strong enough to change participants’ behaviour, because of the high-level standard of care with individual physiotherapy offered to all participants and the higher than expected baseline DEMMI scores.^47 48^ For the mobility goals, we added one level (level 8, walk≥75 m (30 min or stairs) and no bed rest during daytime) to the Johns Hopkins Highest Level of Mobility (JH-HLM) scale because we expected that most patients would qualify for level 7 (walk≥75 m, corresponding to around 100 steps).^33^ Level 8 is in line with studies using goals of 5000 steps or physical activity for more than 30 min, but may not have been clear enough.^35 46 49^ In our study, more than one-third of participants were initially assigned to level 7, but none to level 8. In our experience, patients on level 8 are often quickly discharged, possibly also reflected in the short length of stay of around 5 days.^35 46^ Some study procedures such as measuring time mobilised with a wrist-worn device may have influenced mobility in both arms and thereby reduced differences between groups.^50 51^ Reaching a certain level of mobility may also have triggered discharge from hospital and biased mobilisation time, which did not differ between groups.

We aimed not to use additional resources. Nevertheless, should GDM prove to reduce the length of hospital stay, the deployment of additional personnel could still be deemed cost-effective. In the present study, however, no reduction in length of stay was observed. As most studies employed pre–post designs and that the length of stay is generally decreasing, the beneficial effect of GDM on this outcome remains uncertain.^19 21 35^ Previously, long-term effects of GDM interventions were only seldom investigated with only one study showing a beneficial effect on Life-Space Assessment.^12 21^ With no evidence of short-term effects, it is also not surprising that we did not find any differences in the 3-month outcomes, such as quality of life. The study was also not powered to identify such differences. Remarkably, half of the participants were readmitted or died within 3 months, not only highlighting the vulnerability of the population investigated but also indicating that other factors, particularly comorbidities, may weigh more in terms of severe outcomes.

The study has several strengths. First, we used a randomised design to reduce selection bias and confounding. Potential remaining variation in important baseline characteristics was addressed by adjusting the models for stratification factors and baseline values for differences, which were defined a priori. Thus, some imbalances in certain variables may still be found. Second, outcome assessment was blinded to reduce information bias; however, double blinding was not feasible for our research question. Contamination and performance bias may still have occurred in this single blinded study; for example, nurses and physiotherapists could be treating participants in both groups. However, less than 4% of all admitted patients were included in the study and participants in the control group could only be recognised by wearing the accelerometer, which was also used for other studies, was worn like a watch and did not have any display. The intervention did not directly target nursing staff; nevertheless, the intervention could have further improved the already high level of standard care and thus weakened the observable effect. Third, by including patients from general internal medicine wards, we investigated the intervention in a diverse population of hospitalised patients, for example, in terms of age and DEMMI, thus enhancing external validity. Finally, in contrast to previous studies, we investigated an intervention that does not require additional resources that would not be available in clinical practice.^12^,^1421^

The study also has some limitations, which may explain why we could not detect a clinically meaningful effect of GDM, compared with standard care. First, despite efforts, primary outcome assessment was missing in around 15% of participants. This was mainly a consequence of a shorter-than-expected length of hospital stay in many participants,^30 40 52^ despite having excluded patients with an expected stay of less than 5 days. However, there are no reliable tools to predict length of hospital stay, and analysing the change in physical functioning between admission and hospital discharge would have introduced bias.^53 54^ Second, it is possible that the intervention had uncontrolled effects on the standard-of-care group, for example, by modifying physiotherapist practices. However, the proportion of study participants compared with all patients hospitalised was small and the number of physiotherapists high, making spillover effects unlikely. Third, we assessed adherence by checking electronic health records and presence or absence of the board indicating personal mobility goals.

### Clinical implications and conclusion

In our trial, GDM, which fostered mobility without requiring additional resources that might not be available in everyday clinical practice, did not improve physical functioning or mobilisation time. Subgroup analyses suggest that younger patients and those without previous mobility impairments could benefit from goal setting, which can easily complement routine physiotherapy and be part of an interdisciplinary approach to improve mobility. However, eventually, reinforcement of patient mobility takes time—probably more in those with pre-existing impairments and older patients.

To conclude, improving physical functioning and mobility during an acute medical hospitalisation with a pre-existing high level of care and short lengths of stay remains challenging. Future interventions should target additional barriers that can be modified with resources that are available in everyday practice and investigate how to specifically improve mobility in different subgroups of patients.

## supplementary material

10.1136/bmjopen-2024-086921 online supplemental file 1

10.1136/bmjopen-2024-086921 online supplemental file 2

10.1136/bmjopen-2024-086921 online supplemental file 3

## Data Availability

Data are available on reasonable request.
