# Supplementary material for: Effect of goal-directed mobilisation versus standard care on physical functioning among medical inpatients: the GoMob-in randomised, controlled trial
Source: BMJ Open. 2024 Nov 14;14(11):e086921. doi: 10.1136/bmjopen-2024-086921 (PMC11575328; doi:10.1136/bmjopen-2024-086921)

SUPPLEMENTARY MATERIALS

Supplementary Table 1. Daily mobility goals as indicated on the patient communication boards. 2
Supplementary Table 2. Baseline characteristics of participants. 4
Supplementary Table 3. Hospital parameters. 5
Supplementary Table 4. Number of patients who had some protocol deviations (some patients may have several protocol deviations). 6
Supplementary Table 5. Subgroup analyses of the primary outcome (change in DEMMI score, day 5 - admission) according to age, initial DEMMI score and pre-hospital mobility. 7
Supplementary Figure 1. De Morton Mobility Index (DEMMI) scores of patients receiving standard of care or goal-directed mobilization at baseline and the first follow-up (day 5). Data points above the diagonal line indicate participants with improvements in DEMMI scores. 8
Supplementary Figure 2. Participants' individual tracks of DEMMI score during hospitalization. Days indicate effective measurement of DEMMI scores in the study participants by allocation............9

Supplementary Tables

Supplementary Table 1. Daily mobility goals as indicated on the patient communication boards (in German, English translation below).*

Stufe	Tages-Ziel (mindestens 3 x/Tag)
8	≥ 75 m gehen (30 min oder Treppe) & keine Bettruhe
7	≥ 75 m gehen
6	≥ 7,5 m gehen
5	≥ 10 Schritte gehen
4	≥ 1 Minute stehen
3	Transfer auf den Stuhl / Nachtstuhl
2	An die Bettkante sitzen
1	Aktivitäten im Bett / unselbständiger Transfer

* English translations: Daily mobility goal (at least 3x daily); 8, Walk ≥ 75 m (30 min or stairs) & no bed rest; 7, Walk ≥ 75 m; 6, Walk ≥ 7,5 m; 5, Walk ≥ 10 steps; 4, Wtand ≥ 1 minute; 3, Transfer to a chair or commode; 2, Sit at the edge of the bed; 1 Bed activity / dependent transfer out of the bed

Supplementary Table 2. Baseline characteristics of participants.

	Standard of Care (N = 81)	Goal-directed Mobilization (N = 81)
**Living status**		
**Single**	41 (50.6%)	26 (32.1%)
**With other persons (partner, family, shared flat)**	32 (39.5%)	50 (61.7%)
**Highest education**		
**Mandatory school**	16 (19.8%)	13 (16.2%)
**Professional school**	53 (65.4%)	45 (56.2%)
**Matura**	0 (0.0%)	4 (5.0%)
**College / University**	12 (14.8%)	18 (22.5%)
**Reason for admission**		
**Elective**	7 (8.6%)	5 (6.2%)
**Emergency**	74 (91.4%)	76 (93.8%)
**Intensive care**	5 (6.2%)	10 (12.3%)
**Smoking**	12 (14.8%)	9 (11.1%)
**Self-reported health conditions[#]**		
**Cardiac disease**	33 (40.7%)	41 (50.6%)
**Hypertension**	33 (40.7%)	46 (56.8%)
**Pulmonary disease**	35 (43.2%)	32 (39.5%)
**Diabetes**	18 (22.2%)	19 (23.5%)
**Ulcer**	12 (14.8%)	12 (14.8%)
**Renal disease**	25 (30.9%)	13 (16.0%)
**Liver disease**	12 (14.8%)	10 (12.3%)
**Anemia and other hematological diseases**	21 (25.9%)	19 (23.5%)
**Cancer**	27 (33.3%)	20 (24.7%)
**Depression**	13 (16.0%)	23 (28.4%)
**Osteoarthritis**	38 (46.9%)	34 (42.0%)
**Rheumatoid arthritis**	11 (13.6%)	7 (8.6%)
**Back pain**	36 (44.4%)	44 (54.3%)

[#] According to discharge letters; Self-Administered Comorbidity Questionnaire[46]

Supplementary Table 3. Hospital parameters.

	Standard of Care (N = 81)	Goal-directed Mobilization (N = 81)	*P* value
**Comorbidities**†			
**Cardiac disease**	52 (64.2%)	56 (69.1%)	0.62
**Hypertension**	50 (61.7%)	55 (67.9%)	0.51
**Pulmonary disease**	40 (49.4%)	39 (48.1%)	1.00
**Diabetes**	26 (32.1%)	20 (24.7%)	0.38
**Ulcer**	11 (13.6%)	17 (21.0%)	0.30
**Renal disease**	47 (58.0%)	39 (48.1%)	0.27
**Liver disease**	14 (17.3%)	18 (22.2%)	0.55
**Anaemia and other haematological diseases**	46 (56.8%)	46 (56.8%)	1.00
**Cancer**	30 (37.0%)	23 (28.4%)	0.32
**Depression**	17 (21.0%)	12 (14.8%)	0.41
**Osteoarthritis**	15 (18.5%)	10 (12.3%)	0.38
**Rheumatoid arthritis**	4 (4.9%)	1 (1.2%)	0.37
**Back pain**	10 (12.3%)	16 (19.8%)	0.28

† According to discharge letters structured by the Self-Administered Comorbidity Questionnaire[46]

Supplementary Table 4. Number of patients who had some protocol deviations (some patients may have several protocol deviations).[‡]

	Standard of Care (N = 81)	Goal-directed Mobilization (N = 81)	Total (N = 162)
**Protocol deviation**	22 (27.2%)	18 (22.2%)	40 (24.7%)
**Not eligible**	0 (0.0%)	2 (2.5%)	2 (1.2%)
**Not receiving allocated intervention (cross-overs)**	1 (1.2%)	0 (0.0%)	1 (0.6%)
**Intervention lasted less than 3 days**	17 (21.0%)	10 (12.3%)	27 (16.7%)
**Primary outcome not assessed at day 5 (±2 days)**	20 (24.7%)	16 (19.8%)	36 (22.2%)

[‡] In particular, two patients were enrolled two times in the study. Both were in the Standard of Care group in the first randomization, and in the other arm in the second randomization. Their second entry is a protocol violation, but is still considered in the ITT analysis.

Supplementary Table 5. Subgroup analyses of the primary outcome (change in DEMMI score, day 5 - admission) according to age, initial DEMMI score and pre-hospital mobility.[§]

	**Adjusted difference (95% CI)**	***P* value**
**Age**		
**Younger than 65 years old (N = 46)**	5.0 (-4.5 to 14.5)	0.29
**At least 65 years old (N = 116)**	-1.5 (-6.4 to 3.4)	0.55
**DEMMI at baseline**		
**≤ 40 points (N = 38)**	-3.7 (-14.5 to 7.1)	0.49
**> 40 points (N = 124)**	1.5 (-3.4 to 6.4)	0.53
**Mobility aid at baseline**		
**No (N = 89)**	4.0 (-2.5 to 10.4)	0.22
**Yes (N = 73)**	-3.1 (-9.2 to 3.0)	0.32
Abbreviations: DEMMI, De Morton Mobility Index.		

[§] Differences are adjusted for stratification factors (DEMMI ≤ 40 vs DEMMI > 40 and age < 65 years vs ≥ 65 years); changes from baseline are additionally adjusted for the baseline value.

Supplementary Figures

Supplementary Figure 1. De Morton Mobility Index (DEMMI) scores of patients receiving standard of care or goal-directed mobilization at baseline and the first follow-up (day 5). Data points above the diagonal line indicate participants with improvements in DEMMI scores.

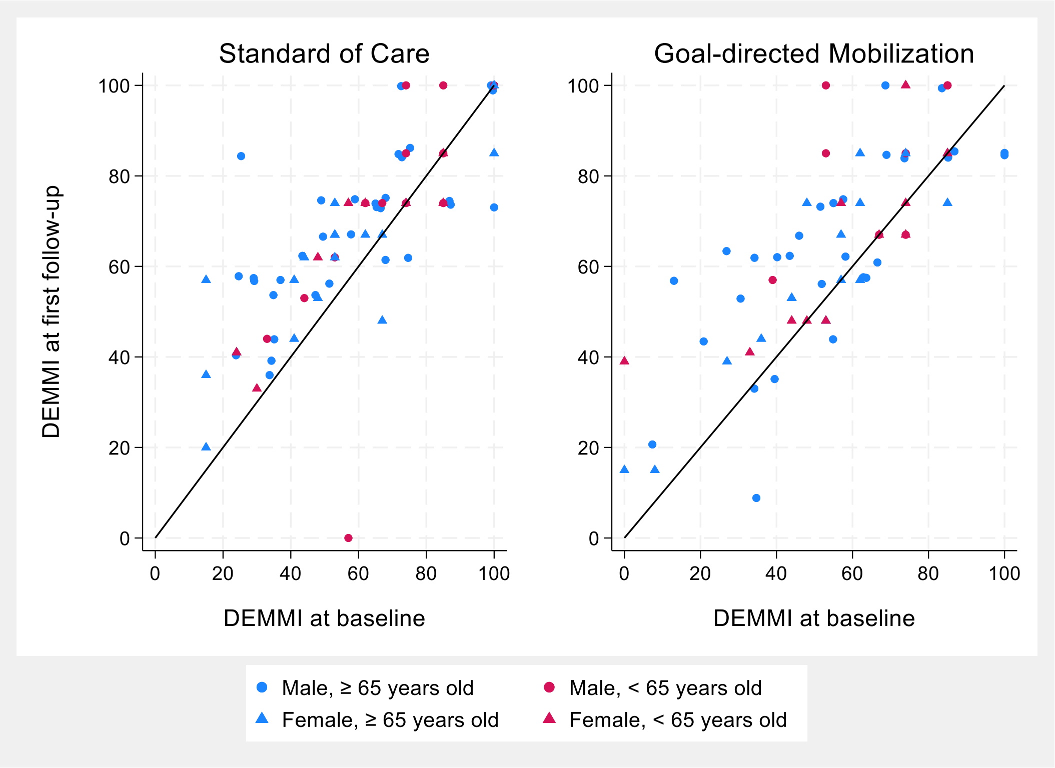

Supplementary Figure 2. Participants' individual tracks of DEMMI score during hospitalization. Days indicate effective measurement of DEMMI scores in the study participants by allocation.

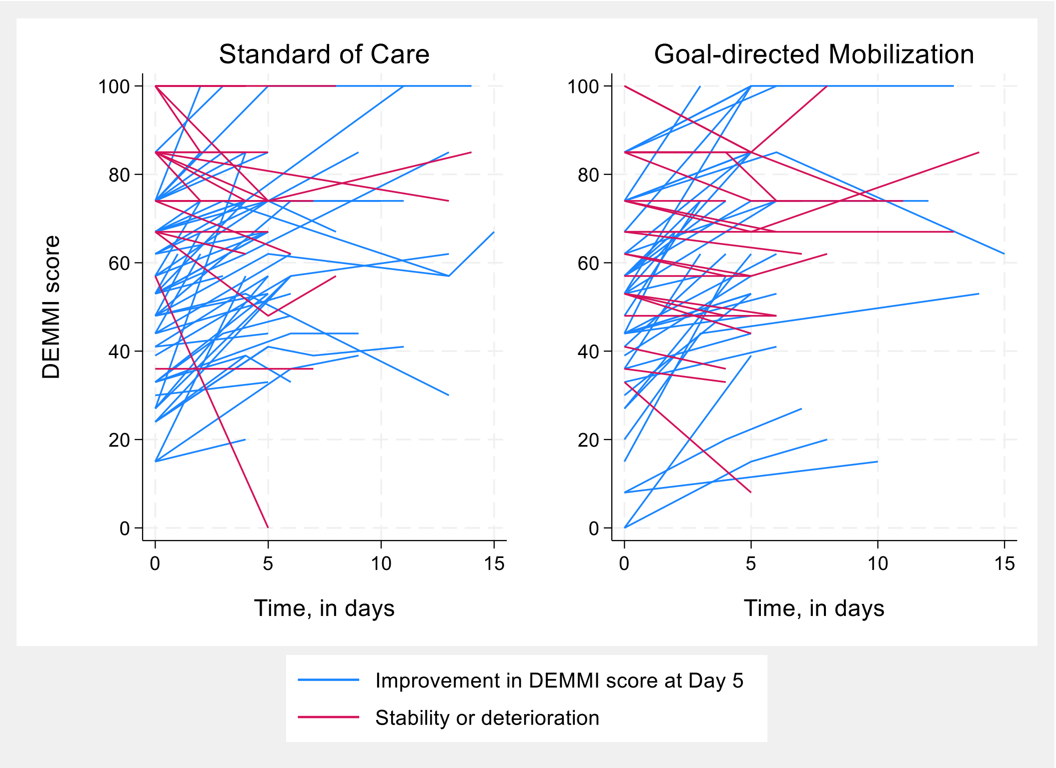

Supplement: online supplemental file 1 [file bmjopen-14-11-s001.pdf]
