# Supplementary material for: Effect of goal-directed mobilisation versus standard care on physical functioning among medical inpatients: the GoMob-in randomised, controlled trial
Source: BMJ Open. 2024 Nov 14;14(11):e086921. doi: 10.1136/bmjopen-2024-086921 (PMC11575328; doi:10.1136/bmjopen-2024-086921)
Supplement: online supplemental file 3 [file bmjopen-14-11-s003.pdf]

Ziel-orientierte Mobilisierung von Spitalpatienten (Goal-directed Mobilization of Medical inpatients, *GoMob-in*)

Patienteninformation

(für die Motivations-Gruppe)[1]

Warum ist es wichtig, mich während dem Spitalaufenthalt zu bewegen?

- Sie können schneller genesen.
- Sie gewinnen Kraft (oder verlieren weniger Kraft).
- Sie bleiben unabhängig.
- Sie behalten Vertrauen in Ihren Körper.
- Das Risiko zu stürzen verringert sich.
- Der Spitalaustritt und die Nachbetreuung wird erleichtert.

Was heisst «frühe Mobilisierung»?

Wenn Sie im Spital sind, sollten Sie sich möglichst viel bewegen (mobilisieren). Sie vermeiden so einen Muskelabbau. Wieviel Sie sich bewegen können, hängt von Ihrem Gesundheitszustand ab. «Früh» heisst, dass Sie sich bereits in den ersten Tagen bewegen sollten, auch wenn Sie sich noch schwach fühlen.

Welche Komplikationen können so verringert werden?

- Wundliegen (Dekubitus)
- Lungen-Entzündung
- Wund-Infekte und Blasenentzündungen
- Blutgerinnsel (Thrombose, Lungenembolie)
- Verstopfung
- Verlust der Muskelkraft mit folglich schlechter Fitness und verringerter Ausdauer

[1] Adaptiert von: Dimitriu M., Early mobilisation in hospital – a guide to help your recovery, Oxford University Hospitals, NHS Foundation Trust, 05/2016, Review 05/2019, https://www.ouh.nhs.uk/patient-guide/leaflets/files/13413Pmobilisation.pdf

- Unsicherheit oder fehlende Selbständigkeit bei Spitalaustritt

Was ist Ziel-orientierte Mobilisierung?

Bei der Ziel-orientierten Mobilisierung wird für jeden Patienten / jede Patientin mit dem Behandlungs-Team ein Ziel festgelegt, wie viel man sich mindestens bewegen soll.

Für diese Studie sind mögliche Ziele in der Tabelle unten vorgegeben.

Die Ziele werden regelmässig angepasst an Ihre Möglichkeiten, zum Beispiel anhand ihrer Krankheit oder ihrer Beweglichkeit.

Stufe	**Tages-Ziel** (mindestens 3 x/Tag)
**8**	**≥ 75 m gehen (30 min oder Treppe) & keine Bettruhe**
**7**	**≥ 75 m gehen**
**6**	**≥ 7,5 m gehen**
**5**	**≥ 10 Schritte gehen**
**4**	**≥ 1 Minute stehen**
**3**	**Transfer auf den Stuhl / Nachtstuhl**
**2**	**An die Bettkante sitzen**
**1**	**Aktivitäten im Bett / unselbständiger Transfer**

Gründe sich zu bewegen

- Bewegung und Aufsitzen verbessert die Durchblutung. Somit verringern Sie das Risiko für Wundliegen, Infektionen und Blutgerinnsel.
- Bewegung hilft, Ihren Magen-Darm-Trakt anzuregen. Dies kann helfen, Übelkeit und Erbrechen entgegenzuwirken. Dabei kann das Risiko für eine Blasenentzündung verringert werden.
- Sitzen und gehen hilft der Lungenfunktion: Schleim und Sekret können besser abgehustet werden. Somit können Sie einer Lungen-Entzündung vorbeugen.
- Sie erhalten Ihre Muskelkraft. Damit behalten oder gewinnen Sie Unabhängigkeit in Ihren Alltagsfunktionen.
- Sie bleiben körperlich und geistig fit und sind somit besser auf die Spitalentlassung vorbereitet.

Schritte für eine frühe Mobilisierung

- Wir unterstützen und motivieren Sie bei der frühen Mobilisierung. Falls Sie Schmerzen haben, erhalten Sie von uns Schmerzmittel.
- Wir legen zusammen mit Ihnen ein Tages-Ziel fest. Die entsprechende Übung sollten Sie mindestens 3x täglich durchführen. Sie dürfen sich gerne auch mehr bewegen.
- Falls Sie Probleme haben oder die Gefahr besteht, dass Sie stürzen, helfen wir Ihnen.
- Falls Sie Hilfsmittel brauchen (Gehstöcke, Rollator) stellen wir Ihnen diese zur Verfügung und instruieren Sie zu deren Gebrauch.
- Falls Sie noch nicht gehen können, zeigen wir Ihnen Übungen, welche Sie im Bett durchführen können.

Was passiert, wenn ich das Spital verlasse?

Wir planen Ihren Spitalaustritt zusammen (Care-Koordinatorin, Pflege, Sozialdienst, ÄrztInnen).

Wir klären mit Ihnen ab, ob Sie direkt nach Hause gehen können (zum Beispiel mit Spitex) oder ob Sie noch weitere Unterstützung benötigen (zum Beispiel Rehabilitation, Pflegebett, etc.).

Haben Sie weitere Fragen?

Falls Sie weitere Fragen *zur Bewegung* haben, wenden Sie sich an Ihr Physiotherapie-Team.

Falls Sie Fragen *zur Studie* haben:

Dr. med. Fabian D. Liechti, MD-PhD
Klinik für Allgemeine Innere Medizin KAIM
Inselspital / Universität Bern
Freiburgstrasse, 3010 Bern
fabian.liechti@insel.ch
Tel. +41 31 632 21 11 / Sucher: 181-7253
