# Supplementary material for: Effect of goal-directed mobilisation versus standard care on physical functioning among medical inpatients: the GoMob-in randomised, controlled trial
Source: BMJ Open. 2024 Nov 14;14(11):e086921. doi: 10.1136/bmjopen-2024-086921 (PMC11575328; doi:10.1136/bmjopen-2024-086921)
Supplement: Supplementary file 4 [file bmjopen-2024-086921.reviewer_comments.pdf]

# PEER REVIEW HISTORY

BMJ Open publishes all reviews undertaken for accepted manuscripts. Reviewers are asked to complete a checklist review form and are provided with free text boxes to elaborate on their assessment. These free text comments are reproduced below.

## ARTICLE DETAILS

### Title (Provisional)

Effect of Goal-directed Mobilisation versus standard care on physical functioning among medical inpatients – The GoMob-in randomised, controlled trial

### Authors

Liechti, Fabian Dominik; Heinzmann, Jeannelle; Schmutz, Nina A; Rossen, Michael L; Rossel, Jean-Benoît; Limacher, Andreas; Schmidt Leuenberger, Joachim M; Baumgartner, Christine; Wertli, Maria M; Aujesky, Drahomir; Verra, Martin; Aubert, Carole Elodie

---

## VERSION 1 - REVIEW

---

| | |
|---|---|
| **Reviewer** | **1** |
| **Name** | **Wei, Ke** |
| **Affiliation** | **Chongqing Medical University First Affiliated Hospital** |
| **Date** | **22-Apr-2024** |
| **COI** | **None** |

---

The present study concluded that effect of Goal-directed Mobilization versus standard care on physical activity among medical inpatients. This a carefully done study and the findings are of considerable interest. Some minor revisions are listed below:

1.The article mentions that 123 participants completed the study out of an initial sample of 162. There is no detailed explanation on how missing data were handled. It is recommended that the authors clearly describe how missing data were addressed, such as whether multiple imputation methods were used, and whether the potential impact of this approach was considered in the statistical analysis.

2.Statistical Significance of Secondary Outcomes:

Although the primary outcome (change in DEMMI score) did not show statistically significant differences, there appears to be limited discussion on the secondary outcomes. The authors should elaborate on why these secondary outcomes did not achieve statistical significance, whether this relates to the study design, sample size, or other experimental conditions.

3.Insufficient Description of Intervention Measures:

The article provides a rather general description of the intervention measures (GDM program), such as "personalized goal setting" and "brief patient education". More detailed information is recommended, including specific content of the education, frequency, and duration of the intervention, which will help readers better understand and replicate the study methodology.

4. Balance of Baseline Characteristics:

The article notes differences between groups at baseline in terms of age and reported depression symptoms. It is advisable to adjust for these potential confounders in the statistical analysis to ensure the reliability of the results. Additionally, a more detailed discussion of how these baseline imbalances may have impacted the study outcomes should be included.

---

| | |
|---|---|
| **Reviewer** | **2** |
| **Name** | **Lin, Yanjuan** |
| **Affiliation** | **Fujian Medical University Union Hospital** |
| **Date** | **27-May-2024** |
| **COI** | **Yes.** |

---

This manuscript explores the impact of goal-directed mobilization on inpatient physical activity, but the intervention program lacks specificity and reference value for clinical practice. Among them, some problems in the paper need to be paid attention to:

1.The research objects selected in this study are hospitalized patients, the research objects are too broad, and the intervention plan implemented lacks particularity.

2.In the part of introduction, there is insufficient elaboration on the goal-directed mobilization intervention. When is the best time to start goal-directed mobilization intervention for hospitalized patients, and is there any debate about the timing and frequency of intervention? If the authors can't answer, it's hard not to doubt the need for the study.

3. In the part of methods, what is the sample size of this study calculated according to? How many cases are expected to be collected in the control group and the trial group?

4. In this study, was there a safety assessment before implementing the intervention? Is there a unified theoretical and practical training for the participants? Do the working qualifications of the participants meet the requirements of the intervention? How to judge the quality of intervention? Are there any activity termination criteria? It's all confusing.

5.Does the intervention plan have a specific intervention time, frequency and cycle, and is there a specific intervention process?

6. In the discussion section, analyses of secondary outcomes are missing. Studies showing that interventions are associated with shorter hospital stays and improved quality of life after discharge are not discussed.

Based on the above opinions, the overall quality of the paper is poor, and it is recommended to reject the paper.

---

| | |
|---|---|
| **Reviewer** | **3** |
| **Name** | **Valkenet, Karin** |
| **Affiliation** | **University Medical Centre Utrecht** |
| **Date** | **05-Jul-2024** |
| **COI** | **no competing interests.** |

---

The authors present a relevant and well written and well reported article.

However, I have some majors concerns about the design of the intervention and the contrasts between the groups.

Majors issues:

In your discussion you mention shortly that your intervention may not have been strong enough to change participants behaviour. I think this is the major limitation of your study and I think this should get more attention in your discussion and interpretation. Walking 75 meters is the highest goal setting level in your intervention. This is about 100 steps / 2 minutes. In my opinion this is not a challenging goal. In the pre-post study of Grootel et al. on the impact of a GDM intervention (PMCID: PMC10492426) the average minutes of PA in hospital at baseline was 38 minutes which improved to 50 minutes. In the RCT of Yamashita et al. (PMCID: PMC10765285) they use a step goal of 5000 steps per day in hospital which resulted in almost a twofold increase of PA in the intervention group. Finally, the review of Gallardo-Gomez et al. (PMID: 37536984) says a minimum of PA of 40 minutes during hospital stay is necessary to improve Physical Functioning. That is a big difference compared to 3x times per day 75 meters (which is how I interpreted your intervention). Based on these studies, do the authors think the used GDM intervention was progressive enough to actually improve physical functioning levels of inpatients?

I also have doubts on the possible spill-over effect. Based on your sentence ‘we dropped the criterion of only including one patient per room’ I assumed that there could be multiple patients per room. The authors also describe that all participants were equipped with a wrist-worn accelerometer (GENEActiv) to measure physical activity (see paragraph participants and sample size). This by itself might be considered an intervention. But my main concern is that blinding was not really the case as everybody could see who was wearing the GENEActiv. Furthermore, the identified individual mobility goal was depicted on a patient communication board next to the bed so everybody could also dee who was in the

intervention group. Furthermore, the goal on the white board influences patients in same room, but also health care providers and family as they can see the goal of the other patient. In the discussion the authors mention that the risks of spill-over-effects was small as physio's, but I am not convinced. Could you please comment on this?

Minor issues:

You define the DEMMI as a tool that measures Physical Activity. I don't agree, I think it measures Physical Functioning.

The actual goal setting method of the intervention remained a little vague to me. It is described in text but I think Table 2 from the appendix is more clear. Maybe add this in the text. And please elaborate more on how/how often the goal was evaluated and adapted. The contrast with the control group is not very clear to me.

The authors state 'patient communication boards indicating mobility goals were removed' > I don't understand this. This is the main part of your intervention and contrast with the control group; why would you remove this?

Paragraph participants and sample size > I miss the sample size calculation (I see it's in another paragraph)

Sample size calculation: difference in change … 5 score points > why was this number chosen? Is this a known minimal clinical detectable change for example? I see references, but please give short information on this in the text.

Could you elaborate more on the GENEActiv. Was the amount of PA visible to the participants for example?

You mention the RCT as a strength. I have doubts that in this case it is a strength as spill-over between I and C seems very easily done without you knowing it (see my main concern earlier).

---

## VERSION 1 - AUTHOR RESPONSE

## Reviewer 1 Comments

Dr. Ke Wei, Chongqing Medical University First Affiliated Hospital

Comments to the Author:

The present study concluded that effect of Goal-directed Mobilization versus standard care on physical activity among medical inpatients. This a carefully done study and the findings are of considerable interest. Some minor revisions are listed below:

## Reviewer 1 – comment 1

The article mentions that 123 participants completed the study out of an initial sample of 162. There is no detailed explanation on how missing data were handled. It is recommended that the authors clearly describe how missing data were addressed, such as whether multiple imputation methods were used, and whether the potential impact of this approach was considered in the statistical analysis.

## Response to reviewer 1 – comment 1

*We used multiple imputation by chained equation. This information was added to the Statistical Analysis section (p 9):*

> *"If outcomes were missing, we employed multiple imputation in the primary analysis, including the subgroup analyses, except for categorical and count outcomes, where only age, sex and BMI were used in the imputation model. We used multiple imputation by chained equation to impute all variables separately, generating fifty imputed data set as predefined in the statistical analysis plan (Supplementary materials). In a secondary per-protocol analysis, without using imputation for missing values, patients that violated any eligibility criteria, did not receive the allocated intervention, or were discharged before day 4, were disregarded. "*

## Reviewer 1 – comment 2

Statistical Significance of Secondary Outcomes:

Although the primary outcome (change in DEMMI score) did not show statistically significant differences, there appears to be limited discussion on the secondary outcomes. The authors should elaborate on why these secondary outcomes did not achieve statistical significance, whether this relates to the study design, sample size, or other experimental conditions.

## Response to reviewer 1 – comment 2

*Thank you for giving us the opportunity to discuss the secondary outcomes in more details in the Discussion, which we have restructured. The sample size was based on the primary outcome; hence, for many secondary outcomes, the study may indeed be underpowered. We have added the following paragraphs to the Discussion (p 16):*

> *"In our experience, patients on level 8 are often quickly discharged, possibly also reflected in the short length of stay of around 5 days. (van Grootel et al. 2023; Gallardo-Gomez et al. 2023) Reaching a certain level of mobility may also have triggered discharge from hospital and biased mobilisation time, which did not differ between groups.*
>
> *We aimed not to use additional resources. Nevertheless, should GDM prove to reduce the length of hospital stay, the deployment of additional personnel could still be deemed cost-effective. In the present study, however, no reduction in length of stay*

*was observed. As most studies employed pre-post designs and the length of stay is generally decreasing, the beneficial effect of GDM on this outcome remains uncertain. (Heinzmann, Baumgartner, and Liechti 2022; Hoyer et al. 2016; van Grootel et al. 2023)"*

*Previously, long-term effects of GDM interventions were only seldom investigated with only one study showing a beneficial effect on Life-Space Assessment. (Brown et al. 2016; Heinzmann, Baumgartner, and Liechti 2022) With no evidence of short-term effects, it is also not surprising that we did not find any differences in the three-month outcomes, such as quality of life. The study was also not powered to identify such differences. Remarkably, half of the participants were readmitted or died within 3 months, highlighting the vulnerability of the population investigated, but also indicating that other factors, foremost comorbidities, may weigh more in terms of severe outcomes."*

## Reviewer 1 – comment 3
## Insufficient Description of Intervention Measures:

The article provides a rather general description of the intervention measures (GDM program), such as "personalized goal setting" and "brief patient education". More detailed information is recommended, including specific content of the education, frequency, and duration of the intervention, which will help readers better understand and replicate the study methodology.

## Response to reviewer 1 – comment 3

*Thank you for the suggestion. We adapted the paragraph describing the intervention as follows (p 7):*

*"The experimental intervention consisted of a GDM program during hospitalisation. After randomisation, the study team assigned in discussion with the patient the first individual mobility goal. Mobility goals were adapted from the Johns Hopkins Highest Level of Mobility and used 8 levels (1, Bed activities; 2, sit at edge of bed; 3, transfer to chair/commode; 4, stand for > 1min; 5, walk ≥ 10 steps; 6, walk ≥ 7.5m; 7, walk ≥ 75m; 8, walk ≥ 75m [30min or stairs] and no bed rest during daytime). (Hoyer et al. 2018) Participants were instructed to complete the mobility goal at least three times daily. The identified individual mobility goal was then depicted on a patient communication board next to the bed, visible for all stakeholders (patients, visiting friends and family, physicians, nurses, physiotherapists; Supplementary Table 1). Participants in the intervention group received a leaflet with a guide on importance, aims and implementation of early mobilisation during hospitalisation, including definitions of mobility goals (Supplementary materials). During the first physiotherapy session (at latest on the first working day after study inclusion), a*

*trained physiotherapist provided short education on GDM. The physiotherapist revised the individual mobility goal level, in discussion with the patient and taking into account current mobility capacities, comorbidities, and perspectives of recovery. The frequency and total number of physiotherapy sessions was at the discretion of the treating physiotherapists. The number of physiotherapy sessions was retrieved from electronic health records at the end of the study. To avoid using additional resources, i.e. increasing the amount of physiotherapy provided, the intervention focussed on indicating a clear goal to increase mobilisation throughout the hospital stay, particularly between physiotherapy sessions, when no specific staff was available. The intervention was formally stopped at hospital discharge, transfer to a unit other than general internal medicine, or 15 days after study inclusion. Patient communication boards were removed, and goal setting not anymore actively encouraged, but, if necessary, patients would still receive routine physiotherapy. All healthcare professionals caring for the participant were allowed to adapt the mobility goal level together with the patient at any time – usually to a higher level, but level reductions were also allowed."*

*To understand the distribution of the first mobility goal selected, we added the table indicating the first mobility goals to the main manuscript (Table 2, page 12).*

**Reviewer 1 – comment 4**

Balance of Baseline Characteristics:

The article notes differences between groups at baseline in terms of age and reported depression symptoms. It is advisable to adjust for these potential confounders in the statistical analysis to ensure the reliability of the results. Additionally, a more detailed discussion of how these baseline imbalances may have impacted the study outcomes should be included.

**Response to reviewer 1 – comment 4**

***We have identified age and prehospital mobility in the literature as being associated with DEMMI scores. Therefore, we used these variables for stratification in the randomisation and for subgroup analyses, as defined in the published protocol (Liechti et al. 2022) and statistical analysis plan, made available in the appendix. All models are adjusted for stratification factors used at randomisation, changes from baseline and improvements are additionally adjusted for the baseline value. Considering the moderate total number of participants and the large total number of possible variables available for analysis, finding such a potentially***

***spurious imbalance is not surprising, but should primarily be considered at planning of the analysis; hence also the mentioned stratification and adjusting. However, as randomisation was properly done as planned, we don't see the need for additional investigations or model adjusting (Roberts and Torgerson 1999). Therefore, we did not stratify or adjusted by self-reported depression. Also, depression according to the discharge diagnosis was not imbalanced (Supplementary Table 2). To better illustrate this, we have added the full table of baseline characteristics to the supplementary materials (Supplementary Table 1) Furthermore, we have extended the results and discussion sections to respond to the reviewer's concerns:***

> ***"Intervention and control groups were similar at baseline, except for older age and more self-reported depression in the intervention group, but not according to the depression diagnoses reported in the discharge letters." (p 10)***
>
> ***"Potential remaining variation in important baseline characteristics was addressed by adjusting the models for stratification factors and baseline values for differences, which were defined a priori. Thus, some imbalances in some variables imbalances may still be found." (p 16)***

## Reviewer 2 comments

Dr. Yanjuan Lin, Fujian Medical University Union Hospital

Comments to the Author:

This manuscript explores the impact of goal-directed mobilization on inpatient physical activity, but the intervention program lacks specificity and reference value for clinical practice. Among them, some problems in the paper need to be paid attention to:

### Reviewer 2 – comment 1

The research objects selected in this study are hospitalized patients, the research objects are too broad, and the intervention plan implemented lacks particularity.

### Response to reviewer 2 – comment 1

*Studies in more selected patient groups, e.g. patients on intensive care or geriatric units have been conducted (Hoyer et al. 2016; Klein et al. 2018; Schaller et al. 2016). General internal medicine patients however represent the largest, fast growing, and most resource-intensive inpatient group, many of whom suffer from chronic diseases and fatigue, are multimorbid*

*and highly complex (Verma et al. 2017; Nardi et al. 2007; Liechti et al. 2021). It is thus important to understand if goal-directed mobilisation has also positive effects in a broader population, i.e. general internal medicine inpatients.*

*We added the following to the Introduction, to address the reviewer's concerns (p 4):*

> *"Also, the effects of GDM in general internal medicine inpatients – a large, quickly growing, and resource-intensive inpatient group, many of whom suffer from chronic diseases and fatigue and are multimorbid and highly complex – is uncertain.(Verma et al. 2017; Nardi et al. 2007; Liechti et al. 2021)*
>
> *To address previous studies' limitations and the evidence gap regarding GDM in a diverse patient population, we conducted the … GoMob-in trial…"*

### Reviewer 2 – comment 2

In the part of introduction, there is insufficient elaboration on the goal-directed mobilization intervention. When is the best time to start goal-directed mobilization intervention for hospitalized patients, and is there any debate about the timing and frequency of intervention? If the authors can't answer, it's hard not to doubt the need for the study.

### Response to reviewer 2 – comment 2

*Thank you for the question. There is consensus that early mobilisation, started usually within the first 24h after admission, is beneficial in general internal medicine patients and ICU patients. (Bergbower et al. 2020; Schaller et al. 2024; Pashikanti and Von Ah 2012) Therefore, it can be assumed that GDM should also start early during hospitalisation as a mean to improve mobilisation. We have extended the introduction to address the reviewer's issues as follows (p 4):*

> *"GDM is usually started early during hospitalisation or intensive care treatment, where it has been shown to improve physical activity in selected patient groups (Heinzmann, Baumgartner, and Liechti 2022; Bergbower et al. 2020)."*

### Reviewer 2 – comment 3

In the part of methods, what is the sample size of this study calculated according to? How many cases are expected to be collected in the control group and the trial group?

### Response to reviewer 2 – comment 3

*The sample size was calculated according to the minimal clinically important difference reported in previous studies and referenced in the manuscript. We expected to include 128 participants in the control group and 128 participants in the intervention group We expanded the paragraph on sample size calculation to reference directly to the MCID as follows:*

> *"We estimated that a sample size of 64 in each group (total 128 participants) would have a power of 80% to detect a difference in DEMMI change within 5 days of 5 score points, the minimal clinically important difference, with a standard deviation (SD) of 10 points at a two-sided alpha-level of 0.05. (Karlsen et al. 2017; Oestergaard et al. 2018; de Morton, Davidson, and Keating 2008, 2010; Zupanc et al. 2019)" (p 9)*

### Reviewer 2 – comment 4

In this study, was there a safety assessment before implementing the intervention?

### Response to reviewer 2 – comment 4

*A pre-study safety assessment was exempted by the Ethics Committee, because the intervention was considered to be a minimal risk. During the study, the Principal Investigator made a causality assessment of serious adverse events to the trial intervention. Any event assessed as possibly, probably or definitely related to the intervention was classified as related to the trial intervention and reported to the Ethics Committee according to the protocol. We did not encounter any relevant serious adverse events.*

### Reviewer 2 – comment 5

Is there a unified theoretical and practical training for the participants?

### Response to reviewer 2 – comment 5

*There was a unified theoretical training for the participants ("short education"). The participants received an individual practical training by physiotherapists. The physiotherapists received a unified theoretical and practical training. We added the table indicating the first mobility goals to the main manuscript (Table 2, page 12) and adapted the paragraph on how the first mobility goal was set (p 7):*

> *"Participants in the intervention group received a leaflet with a guide on importance, aims and implementation of early mobilisation during hospitalisation, including definitions of mobility goals (Supplementary materials). During the first physiotherapy session (at latest on the first working day after study inclusion), a trained physiotherapist provided short education on GDM."*
>
> *"Trained physiotherapists with experience in general internal medicine were instructed […]"*

### Reviewer 2 – comment 6

Do the working qualifications of the participants meet the requirements of the intervention?

### Response to reviewer 2 – comment 4

*Participants were eligible for participation if they met the requirements described in the Methods (p 5). Training of physiotherapists includes at least a 4-year Bachelor's degree. We have adapted the Methods section as follows (p 7).*

> *"Trained physiotherapists with experience in general internal medicine were instructed at trial inception […]"*

### Reviewer 2 – comment 7

How to judge the quality of intervention?

### Response to reviewer 2 – comment 7

*The intervention was assessed by checking adherence through presence or absence of the Patient Communication Board and compliance of staff by checking entries to the electronic health records. We have highlighted this in the manuscript's Methods and added a paragraph in the Discussion.*

> *"Protocol adherence was assessed at study visits by presence of the board displaying the mobility goal level and by nurses' and physiotherapists' entries to the electronic health record to detect any protocol deviation." (p 7)*
>
> *"Third, we assessed adherence by checking electronic health records and presence or absence of the board indicating personal mobility goals." (p 17)*

### Reviewer 2 – comment 8

Are there any activity termination criteria? It's all confusing.

### Response to reviewer 2 – comment 8

*Yes, there were some termination criteria. To be more precise, we added the following to the Methods section:*

> *"The accelerometers were activated when participants were fitted with it and collected by the study team when discharged from the hospital, transferred to a unit other than general internal medicine for longer than 24 hours, or 15 days after study inclusion (end of the intervention) to read-out the data." (p 6)*
>
> *"The intervention was formally stopped at hospital discharge, transfer to a unit other than general internal medicine, or 15 days after study inclusion." (p 7)*

### Reviewer 2 – comment 9

Does the intervention plan have a specific intervention time, frequency and cycle, and is there a specific intervention process?

## Response to reviewer 2 – comment 9

*The intervention plan did not have any specific intervention time. The first physiotherapy session was scheduled on the day of study inclusion or at latest on the next working day. The frequency was up to the discretion of the treating physiotherapist. The intervention consisted of the short education, supported by a hand-out, and the individual goal setting, but did not include other structured interventions.*

*The paragraph describing the intervention has been restructured and amended to indicate the requested information on the intervention as follows (p 7):*

> *"The experimental intervention consisted of a GDM program during hospitalisation. After randomisation, the study team assigned in discussion with the patient the first individual mobility goal. Mobility goals were adapted from the Johns Hopkins Highest Level of Mobility and used 8 levels (1, Bed activities; 2, sit at edge of bed; 3, transfer to chair/commode; 4, stand for > 1min; 5, walk ≥ 10 steps; 6, walk ≥ 7.5m; 7, walk ≥ 75m; 8, walk ≥ 75m [30min or stairs] and no bed rest during daytime). (Hoyer et al. 2018) Participants were instructed to complete the mobility goal at least three times daily. The identified individual mobility goal was then depicted on a patient communication board next to the bed, visible for all stakeholders (patients, visiting friends and family, physicians, nurses, physiotherapists; Supplementary Table 1). Participants in the intervention group received a leaflet with a guide on importance, aims and implementation of early mobilisation during hospitalisation, including definitions of mobility goals (Supplementary materials). During the first physiotherapy session (at latest on the first working day after study inclusion), a trained physiotherapist provided short education on GDM. The physiotherapist revised the individual mobility goal level, in discussion with the patient and taking into account current mobility capacities, comorbidities, and perspectives of recovery. The frequency and total number of physiotherapy sessions was at the discretion of the treating physiotherapists. The number of physiotherapy sessions was retrieved from electronic health records at the end of the study. To avoid using additional resources, i.e. increasing the amount of physiotherapy provided, the intervention focussed on indicating a clear goal to increase mobilisation throughout the hospital stay, particularly between physiotherapy sessions, when no specific staff was available. The intervention was formally stopped at hospital discharge, transfer to a unit other than general internal medicine, or 15 days after study inclusion. Patient communication boards were removed, and goal setting not anymore actively encouraged, but, if necessary, patients would still receive routine physiotherapy. All healthcare professionals caring for the participant were allowed to adapt the mobility goal level together with the patient at any time – usually to a higher level, but level reductions were also allowed."*

Reviewer 2 – comment 10

In the discussion section, analyses of secondary outcomes are missing. Studies showing that interventions are associated with shorter hospital stays and improved quality of life after discharge are not discussed.

## Response to reviewer 2 – comment 10

*Thank you for giving us the opportunity to discuss the secondary outcomes in more details in the Discussion, which we have restructured. The sample size was based on the primary outcome; hence, for many secondary outcomes the study may indeed be underpowered. We have added the following paragraphs to the Discussion (p 16):*

> *"In our experience, patients on level 8 are often quickly discharged, possibly also reflected in the short length of stay of around 5 days. (van Grootel et al. 2023; Gallardo-Gomez et al. 2023) Reaching a certain level of mobility may also have triggered discharge from hospital and biased mobilisation time, which did not differ between groups.*
>
> *We aimed not to use additional resources. Nevertheless, should GDM prove to reduce the length of hospital stay, the deployment of additional personnel could still be deemed cost-effective. In the present study, however, no reduction in length of stay was observed. As most studies employed pre-post designs and the length of stay is generally decreasing, the beneficial effect of GDM on this outcome remains uncertain. (Heinzmann, Baumgartner, and Liechti 2022; Hoyer et al. 2016; van Grootel et al. 2023)"*
>
> *Previously, long-term effects of GDM interventions were only seldom investigated with only one study showing a beneficial effect on Life-Space Assessment. (Brown et al. 2016; Heinzmann, Baumgartner, and Liechti 2022) With no evidence of short-term effects, it is also not surprising that we did not find any differences in the three-month outcomes, such as quality of life. The study was also not powered to identify such differences. Remarkably, half of the participants were readmitted or died within 3 months, highlighting the vulnerability of the population investigated, but also indicating that other factors, foremost comorbidities, may weigh more in terms of severe outcomes."*

Reviewer 2 – comment 11

Based on the above opinions, the overall quality of the paper is poor, and it is recommended to reject the paper.

## Response to reviewer 2 – comment 11

*We hope the adaptations to address the different comments could improve the quality of our manuscript. We thank all reviewers for the useful and constructive feedback.*

## Reviewer: 3

Dr. Karin Valkenet, University Medical Centre Utrecht

Comments to the Author:

The authors present a relevant and well written and well reported article.

However, I have some majors concerns about the design of the intervention and the contrasts between the groups.

Majors issues:

### Reviewer 3 – comment 1

In your discussion you mention shortly that your intervention may not have been strong enough to change participants behaviour. I think this is the major limitation of your study and I think this should get more attention in your discussion and interpretation. Walking 75 meters is the highest goal setting level in your intervention. This is about 100 steps / 2 minutes. In my opinion this is not a challenging goal. In the pre-post study of Grootel et al. on the impact of a GDM intervention (PMCID: PMC10492426) the average minutes of PA in hospital at baseline was 38 minutes which improved to 50 minutes. In the RCT of Yamashita et al. (PMCID: PMC10765285) they use a step goal of 5000 steps per day in hospital which resulted in almost a twofold increase of PA in the intervention group. Finally, the review of Gallardo-Gomez et al. (PMID: 37536984) says a minimum of PA of 40 minutes during hospital stay is necessary to improve Physical Functioning. That is a big difference compared to 3x times per day 75 meters (which is how I interpreted your intervention). Based on these studies, do the authors think the used GDM intervention was progressive enough to actually improve physical functioning levels of inpatients?

### Response to reviewer 3 – comment 1

*We agree with the reviewer's concerns that the intervention may have been too weak. However, the highest goal was 'walk ≥ 75m [30min or stairs] and no bed rest during daytime'. Also, in setting the first mobility goal levels, no ceiling effect was observed. Therefore, the 'weakness' of the intervention may be less in the goal levels, but in the implementation, which would have needed, e.g. more staff to reinforce, which was against our aim not to use additional resources. It is also possible that hospital stays were too short to have an impact. We have added these remarks to the Discussion as follows (p 15–16).*

*"Second, the time window may have been too short for an effective intervention, for example, in a previous study physical activity increased only after the third day of a GDM intervention while a meta-analysis modelled increasing effectiveness after admission until two weeks after discharge.(Teodoro et al. 2016; Gallardo-Gomez et al. 2023)*

*With the length of hospital stay becoming shorter, the time being physically active (measured as proportion of hospitalisation time) may remain low, despite improving*

*physical activity. (Valkenet et al. 2019) This can be explained by both the patients' enfeeblement due to their presenting complaints and time-consuming investigations and treatments mostly during the beginning of hospitalisation. Nevertheless, 40% of our participants were transferred to another institution (e.g. hospital, rehabilitation centre or a nursing home) after their index hospitalisation, where a goal-setting mobilisation could be continued to improve physical activity.*

*Third, our intervention may not have been strong enough to change participants' behaviour, because of the high-level standard of care with individual physiotherapy offered to all participants and the higher than expected baseline DEMMI scores. (D'Souza et al. 2023; Hartley et al. 2021) For the JH-HLM, we added one level (level 8, walk ≥ 75m [30min or stairs] and no bed rest during daytime) because we expected that most patients would qualify for level 7 (walk ≥ 75m, corresponding to around 100 steps). (Hoyer et al. 2018) Level 8 is in line with studies using goals of 5,000 steps or physical activity. (Gallardo-Gomez et al. 2023; van Grootel et al. 2023; Yamashita et al. 2023) In our study, more than a third of participants were initially assigned to level 7, but none to level 8. In our experience, patients level 8 are often quickly discharged, possibly also reflected in the short length of stay of around 5 days. (van Grootel et al. 2023; Gallardo-Gomez et al. 2023) Reaching a certain level of mobility may also have triggered discharge from hospital and biased mobilisation time, which did not differ between groups.*

*We aimed not to use additional resources. Nevertheless, should GDM prove to reduce the length of hospital stay, the deployment of additional personnel could still be deemed cost-effective. In the present study, however, no reduction in length of stay was observed. As most studies employed pre-post designs and that the length of stay is generally decreasing, the beneficial effect of GDM on this outcome remains uncertain. (Heinzmann, Baumgartner, and Liechti 2022; Hoyer et al. 2016; van Grootel et al. 2023)"*

## Reviewer 3 – comment 2

I also have doubts on the possible spill-over effect. Based on your sentence 'we dropped the criterion of only including one patient per room' I assumed that there could be multiple patients per room. The authors also describe that all participants were equipped with a wrist-worn accelerometer (GENEActiv) to measure physical activity (see paragraph participants and sample size). This by itself might be considered an intervention. But my main concern is that blinding was not really the case as everybody could see who was wearing the GENEActiv. Furthermore, the identified individual mobility goal was depicted on a patient communication board next to the bed so everybody could also dee who was in the intervention group. Furthermore, the goal on the white board influences patients in same room, but also health care providers and family as they can see the goal of the other patient.

In the discussion the authors mention that the risks of spill-over-effects was small as physio's, but I am not convinced. Could you please comment on this?

## Response to reviewer 3 – comment 2

*We did not consider the accelerometers to be an intervention and therefore opted for a device not giving feedback to participants or staff.*

> *"The accelerometer has no display because the study's aim was to evaluate implementation of GDM without using additional technological assets, which has already been proven to function, but comes at additional costs. (van Grootel et al. 2023)" (p 6)*

*This was a single blinded trial with the primary outcome assessors being blinded. As the patients were not fully blinded, we can not exclude some spill-over effects of the intervention. To reduce contamination between the intervention and control group, we initially allowed only one participant per patient room. Throughout the study, we realised, that this criterion was not a good way to separate participants from the two groups, but hampered recruitment and we decided to drop the criterion. To address the reviewer's concerns of contamination, we added some explanations to the Methods, Results, and Discussion.*

> *"Compared to the original protocol, we dropped the criterion of only including one patient per room after March 2022 to increase recruitment because patients were often moved to other rooms and staff was not attributed to the same rooms each following day." (p 5)*
>
> *"[…] we enrolled 162 patients among 5,744 admissions to our department […]" (p 10)*
>
> *"Contamination and performance bias may still have occurred in this single blinded study; for example, nurses and physiotherapists could be treating participants in both groups. However, less than 4% of all admitted patients were included in the study and participants in the control group could only be recognised by wearing the accelerometer, which was also used for other studies, was worn like a watch, and did not have any display. The intervention did not directly target nursing staff; nevertheless, the intervention could have further improved the already high level of standard care and thus weakened the observable effect." (p 16–17)*
>
> *"[…] our intervention may not have been strong enough to change participants' behaviour, because of the high-level standard of care with individual physiotherapy offered to all participants in our hospital and and the higher than expected baseline DEMMI scores. (D'Souza et al. 2023; Hartley et al. 2021)" (p 15–16)*

Minor issues:

## Reviewer 3 – comment 3

You define the DEMMI as a tool that measures Physical Activity. I don't agree, I think it measures Physical Functioning.

## Response to reviewer 3 – comment 3

*For more consistency, we have adapted the manuscript to use "Physical Functioning" throughout when referring to the DEMMI score measures and "Mobilisation Time" when referring to the physical activity as measured by accelerometers in the present study.*

## Reviewer 3 – comment 4

The actual goal setting method of the intervention remained a little vague to me. It is described in text but I think Table 2 from the appendix is more clear. Maybe add this in the text. And please elaborate more on how/how often the goal was evaluated and adapted. The contrast with the control group is not very clear to me.

## Response to reviewer 3 – comment 4

*We added the table indicating the first mobility goals to the main manuscript (Table 2, page 12) and adapted the paragraph on how the first mobility goal was set (p 6–7):*

> *"After randomisation, the study team assigned in discussion with the patient the first individual mobility goal. Mobility goals were adapted from the Johns Hopkins Highest Level of Mobility and used 8 levels (1, Bed activities; 2, sit at edge of bed; 3, transfer to chair/commode; 4, stand for > 1min; 5, walk ≥ 10 steps; 6, walk ≥ 7.5m; 7, walk ≥ 75m; 8, walk ≥ 75m [30min or stairs] and no bed rest during daytime). (Hoyer et al. 2018) Participants were instructed to complete the mobility goal at least three times daily. The identified individual mobility goal was then depicted on a patient communication board next to the bed, visible for all stakeholders (patients, visiting friends and family, physicians, nurses, physiotherapists; Supplementary Table 1). Participants in the intervention group received a leaflet with a guide on importance, aims and implementation of early mobilisation during hospitalisation, including definitions of mobility goals (Supplementary materials). During the first physiotherapy session (at latest on the first working day after study inclusion), a trained physiotherapist provided short education on GDM. The physiotherapist revised the individual mobility goal level, in discussion with the patient and taking into account current mobility capacities, comorbidities, and perspectives of recovery. The frequency and total number of physiotherapy sessions was at the discretion of the treating physiotherapists. The number of physiotherapy sessions was retrieved from electronic health records at the end of the study. To avoid using additional resources, i.e. increasing the amount of physiotherapy provided, the intervention focussed on indicating a clear goal to increase mobilisation throughout the hospital stay, particularly between physiotherapy sessions, when no specific staff was*

*available. The intervention was formally stopped at hospital discharge, transfer to a unit other than general internal medicine, or 15 days after study inclusion. Patient communication boards were removed, and goal setting not anymore actively encouraged, but, if necessary, patients would still receive routine physiotherapy. All healthcare professionals caring for the participant were allowed to adapt the mobility goal level together with the patient at any time – usually to a higher level, but level reductions were also allowed."*

### Reviewer 3 – comment 5

The authors state 'patient communication boards indicating mobility goals were removed' > I don't understand this. This is the main part of your intervention and contrast with the control group; why would you remove this?

### Response to reviewer 3 – comment 5

*The Patient Communication Boards were only removed during assessment to improve blinding. We have adapted the paragraph accordingly (p 8):*

> *"Shortly before the assessment, patients were instructed not to reveal their group allocation; patient communication boards indicating mobility goals were then removed by nursing staff for the time of the assessment by the physiotherapist to minimise the risk of revealing group allocation to the assessor."*

### Reviewer 3 – comment 6

Paragraph participants and sample size > I miss the sample size calculation (I see it's in another paragraph)

### Response to reviewer 3 – comment 6

*We adapted the heading to "Participants" to fit the content (p 5) and moved the paragraph on sample size calculation to the beginning of the paragraph on Statistical Analysis.*

### Reviewer 3 – comment 7

Sample size calculation: difference in change … 5 score points > why was this number chosen? Is this a known minimal clinical detectable change for example? I see references, but please give short information on this in the text.

### Response to reviewer 3 – comment 7

*The sample size was calculated according to the MCID reported in previous studies and referenced in the manuscript. We expected to include 128 participants in the control group*

*and 128 participants in the intervention group We expanded the paragraph on sample size calculation to reference directly to the MCID as follows.*

> *"We estimated that a sample size of 64 in each group (total 128 participants) would have a power of 80% to detect a difference in DEMMI change within 5 days of 5 score points, the minimal clinically important difference, with a standard deviation (SD) of 10 points at a two-sided alpha-level of 0.05" (p 9)*

### Reviewer 3 – comment 8

Could you elaborate more on the GENEActiv. Was the amount of PA visible to the participants for example?

### Response to reviewer 3 – comment 8

*We added the following information to the manuscript (p 6):*

> *"All participants were equipped with a waterproof, wrist-worn accelerometer (GENEActiv) to measure three axes of acceleration at a frequency of 50Hz, allowing uninterrupted data collection for 15 days, along with temperature to verify that the device was worn continuously. (Esliger et al. 2011) The accelerometer has no display because the study's aim was to evaluate implementation of GDM without using additional technological assets, which has already been proven to function, but comes at additional costs. (Cohen et al. 2019; van Grootel et al. 2023) The accelerometers were activated when participants were fitted with it and collected by the study team when discharged from the hospital, transferred to a unit other than general internal medicine for longer than 24 hours, or 15 days after study inclusion (end of the intervention) to read-out the data. Data were not analysed until they had been fully collected from all participants."*

### Reviewer 3 – comment 9

You mention the RCT as a strength. I have doubts that in this case it is a strength as spill-over between I and C seems very easily done without you knowing it (see my main concern earlier).

### Response to reviewer 3 – comment 9

*The randomisation process is helpful in avoiding selection bias and confounding. However, we recognise the reviewer's concerns and address this in the Discussion, which we have adapted as follows (p 16):*

> *"The study has several strengths. First, we used a randomised design to reduce selection bias and confounding. Potential remaining variation in important baseline characteristics was addressed by adjusting the models for stratification factors and*

*baseline values for differences, which were defined a priori*. ***Thus, some imbalances in some variables may still be found.*** *Second, outcome assessment was blinded to reduce information bias; however, double blinding was not feasible for our research question and some attrition bias may still have occurred."f*

## References


Bergbower, E. A. S., C. Herbst, N. Cheng, A. Aversano, K. Pasqualini, C. Hartline, D. Hamby-Finkelstein, C. Brewer, S. Benko, and J. Fuscaldo. 2020. 'A novel early mobility bundle improves length of stay and rates of readmission among hospitalized general medicine patients', *J Community Hosp Intern Med Perspect*, 10: 419-25.

Brown, C. J., K. T. Foley, J. D. Lowman, Jr., P. A. MacLennan, J. Razjouyan, B. Najafi, J. Locher, and R. M. Allman. 2016. 'Comparison of Posthospitalization Function and Community Mobility in Hospital Mobility Program and Usual Care Patients: A Randomized Clinical Trial', *JAMA Intern Med*, 176: 921-7.

Cohen, Y., A. Zisberg, Y. Chayat, N. Gur-Yaish, E. Gil, C. Levin, D. Rand, and M. Agmon. 2019. 'Walking for Better Outcomes and Recovery: The Effect of WALK-FOR in Preventing Hospital-Associated Functional Decline Among Older Adults', *J Gerontol A Biol Sci Med Sci*, 74: 1664-70.

D'Souza, A. N., C. L. Granger, J. E. Kay, and C. M. Said. 2023. 'Physical activity is low before and during hospitalisation: A secondary observational study in older Australian general medical patients', *Australas J Ageing*, 42: 545-53.

de Morton, N. A., M. Davidson, and J. L. Keating. 2008. 'The de Morton Mobility Index (DEMMI): an essential health index for an ageing world', *Health Qual Life Outcomes*, 6: 63.

———. 2010. 'Validity, responsiveness and the minimal clinically important difference for the de Morton Mobility Index (DEMMI) in an older acute medical population', *BMC Geriatr*, 10: 72.

Esliger, D. W., A. V. Rowlands, T. L. Hurst, M. Catt, P. Murray, and R. G. Eston. 2011. 'Validation of the GENEA Accelerometer', *Med Sci Sports Exerc*, 43: 1085-93.

Gallardo-Gomez, D., J. Del Pozo-Cruz, H. Pedder, R. M. Alfonso-Rosa, F. Alvarez-Barbosa, M. Noetel, U. Jasper, S. Chastin, J. Ramos-Munell, and B. Del Pozo Cruz. 2023. 'Optimal dose and type of physical activity to improve functional capacity and minimise adverse events in acutely hospitalised older adults: a systematic review with dose-response network meta-analysis of randomised controlled trials', *Br J Sports Med*, 57: 1272-78.

Hartley, P., R. Romero-Ortuno, I. Wellwood, and C. Deaton. 2021. 'Changes in muscle strength and physical function in older patients during and after hospitalisation: a prospective repeated-measures cohort study', *Age Ageing*, 50: 153-60.

Heinzmann, J., C. Baumgartner, and F. D. Liechti. 2022. 'Goal-Directed Mobility of Medical Inpatients-A Mini Review of the Literature', *Front Med (Lausanne)*, 9: 878031.

Hoyer, E. H., M. Friedman, A. Lavezza, K. Wagner-Kosmakos, R. Lewis-Cherry, J. L. Skolnik, S. P. Byers, L. Atanelov, E. Colantuoni, D. J. Brotman, and D. M. Needham. 2016. 'Promoting mobility and reducing length of stay in hospitalized general medicine patients: A quality-improvement project', *J Hosp Med*, 11: 341-7.

Hoyer, E. H., D. L. Young, L. M. Klein, J. Kreif, K. Shumock, S. Hiser, M. Friedman, A. Lavezza, A. Jette, K. S. Chan, and D. M. Needham. 2018. 'Toward a Common Language for Measuring Patient Mobility in the Hospital: Reliability and Construct Validity of Interprofessional Mobility Measures', *Phys Ther*, 98: 133-42.

Karlsen, A., M. R. Loeb, K. B. Andersen, K. J. Joergensen, F. U. Scheel, I. F. Turtumoeygard, A. L. R. Perez, M. Kjaer, and N. Beyer. 2017. 'Improved Functional Performance in Geriatric Patients During Hospital Stay', *Am J Phys Med Rehabil*, 96: e78-e84.

Klein, L. M., D. Young, D. Feng, A. Lavezza, S. Hiser, K. N. Daley, and E. H. Hoyer. 2018. 'Increasing patient mobility through an individualized goal-centered hospital mobility program: A quasi-experimental quality improvement project', *Nurs Outlook*, 66: 254-62.

Liechti, F. D., T. Beck, A. Ruetsche, M. C. Roumet, A. Limacher, T. Tritschler, and J. D. Donze. 2021. 'Development and validation of a score to assess complexity of general internal medicine patients at hospital discharge: a prospective cohort study', *BMJ Open*, 11: e041205.

Liechti, F. D., J. Heinzmann, J. M. Schmidt Leuenberger, A. Limacher, M. M. Wertli, and M. L. Verra. 2022. 'Effect of goal-directed mobilisation intervention compared with standard care on physical activity among medical inpatients: protocol for the GoMob-in randomised controlled trial', *BMJ Open*, 12: e058509.

Nardi, R., G. Scanelli, S. Corrao, I. Iori, G. Mathieu, and R. Cataldi Amatrian. 2007. 'Co-morbidity does not reflect complexity in internal medicine patients', *Eur J Intern Med*, 18: 359-68.

Oestergaard, A. S., M. H. Mathiesen, A. Karlsen, I. F. Turtumoeygaard, J. Vahlgren, M. Kjaer, and N. Beyer. 2018. 'In acutely admitted geriatric patients, offering increased physical activity during hospitalization decreases length of stay and can improve mobility', *TRANSLATIONAL SPORTS MEDICINE*, 1: 46-53.

Pashikanti, L., and D. Von Ah. 2012. 'Impact of early mobilization protocol on the medical-surgical inpatient population: an integrated review of literature', *Clin Nurse Spec*, 26: 87-94.

Roberts, C., and D. J. Torgerson. 1999. 'Understanding controlled trials: baseline imbalance in randomised controlled trials', *BMJ*, 319: 185.

Schaller, S. J., M. Anstey, M. Blobner, T. Edrich, S. D. Grabitz, I. Gradwohl-Matis, M. Heim, T. Houle, T. Kurth, N. Latronico, J. Lee, M. J. Meyer, T. Peponis, D. Talmor, G. C. Velmahos, K. Waak, J. M. Walz, R. Zafonte, M. Eikermann, and Soms-guided Mobilization Research Initiative International Early. 2016. 'Early, goal-directed mobilisation in the surgical intensive care unit: a randomised controlled trial', *Lancet*, 388: 1377-88.

Schaller, S. J., F. T. Scheffenbichler, T. Bein, M. Blobner, J. J. Grunow, U. Hamsen, C. Hermes, A. Kaltwasser, H. Lewald, P. Nydahl, A. Reisshauer, L. Renzewitz, K. Siemon, T. Staudinger, R. Ullrich, S. Weber-Carstens, H. Wrigge, D. Zergiebel, and S. M. Coldewey. 2024. 'Guideline on positioning and early mobilisation in the critically ill by an expert panel', *Intensive Care Med*.

Teodoro, C. R., K. Breault, C. Garvey, C. Klick, J. O'Brien, T. Purdue, A. Stolaronek, H. W. Wilbur, and L. Matney. 2016. 'STEP-UP: Study of the Effectiveness of a Patient Ambulation Protocol', *Medsurg Nurs*, 25: 111-6.

Valkenet, K., P. Bor, L. van Delft, and C. Veenhof. 2019. 'Measuring physical activity levels in hospitalized patients: a comparison between behavioural mapping and data from an accelerometer', *Clin Rehabil*, 33: 1233-40.

van Grootel, J., P. Bor, J. A. Netjes, C. Veenhof, and K. Valkenet. 2023. 'Improving physical activity in hospitalized patients: The preliminary effectiveness of a goal-directed movement intervention', *Clin Rehabil*, 37: 1501-09.

Verma, A. A., Y. Guo, J. L. Kwan, L. Lapointe-Shaw, S. Rawal, T. Tang, A. Weinerman, P. Cram, I. A. Dhalla, S. W. Hwang, A. Laupacis, M. M. Mamdani, S. Shadowitz, R. Upshur, R. J. Reid, and F. Razak. 2017. 'Patient characteristics, resource use and outcomes associated with general internal medicine hospital care: the General Medicine Inpatient Initiative (GEMINI) retrospective cohort study', *CMAJ Open*, 5: E842-E49.

Yamashita, M., K. Kamiya, N. Hamazaki, S. Uchida, T. Noda, E. Maekawa, and J. Ako. 2023. 'Effects of Acute Phase Intensive Physical Activity (ACTIVE-PA) Monitoring and Education for Cardiac Patients: Pilot Study of a Randomized Controlled Trial', *J Med Internet Res*, 25: e42235.

Zupanc, A., G. Vidmar, P. Novak, and U. Puh. 2019. 'Feasibility of de Morton Mobility Index for adult patients of all ages at low and basic functioning level: a study using the Slovenian translation', *Int J Rehabil Res*, 42: 352-57.

---

## VERSION 2 - REVIEW

| | |
|---|---|
| **Reviewer** | **3** |
| **Name** | **Valkenet, Karin** |
| **Affiliation** | **University Medical Centre Utrecht** |
| **Date** | **29-Aug-2024** |
| **COI** | **no competing interests** |

Thank you for the revisions. The intervention and contrast with the control group is more clear now and the honest discussion about the possible impact of the intervention is appreciated. I have some final suggestions to consider.

Medior suggestion:

- If possible, it would be interesting to see how many patients actually reached their goal, based on the accelerometer data. Do you have any insight in that? For example, in case you find that majority of patients in level 8 do not reach the goal of 30 minutes, that might help explain the negative outcome. And/or when you do not see any difference between I and C, than you know that the intervention did not promote mobilization and therefore did not improve physical functioning.

Minor suggestions:

- Did patient know what the GENEActiv measured? Is it thinkable that just knowing that their physical activity is measured might have resulted in an increase in PA in both groups? This might be a suitable topic for the discussion section.

- As GENEActiv is not part of the intervention, so I would suggest to put the description somewhere else in the manuscript (eg. outcome measurement paragraph)

- There is a discrepancy of sample sizes in the manuscript and the response to the reviewers. In the latter the authors state 'We expected to include 128 participants in the control group and 128 participants in the intervention group' . That does not seem to match with the sample size decription.

- I find level 8 confusing. Walking 30 min or more at a speed of 2.0 km/h means a distance of 1000 meter. That is a big difference with 'walk more than 75 meter' . And also walk a stairs is completely different than walking 30 minutes. Furthermore the difference with level 7 is also confusing as this level is also 'walk more than 75 meter'. There is nothing that you can change about this now, but my experience is that very clear goal setting increases adherence. So this may have hampered adherence, and therefore outcomes.

7 Walk ≥ 75m 29 (35.8%)

8 Walk ≥ 75m (30 min or stairs) 0 (0%)

- First sentence of clinical implications paragraph in discussion section you replaced physical activity with mobilization time. Physical functioning is your primary outcome measure so activity should probably be replaced with functioning.

- In line with previous comment: In that same paragraph you say 'To conclude, improving mobility…' while the DEMMI (ie physical functioning) was your primary outcome. So again I think this should be replaced by physical functioning, or extended to 'physical functioning and mobility' . Please check the correct use of these terms throughout the manuscript.

---

## VERSION 2 - AUTHOR RESPONSE

# Reviewer 3 Comments

Dr. Karin Valkenet, University Medical Centre Utrecht
Comments to the Author:
Thank you for the revisions. The intervention and contrast with the control group is more clear now and the honest discussion about the possible impact of the intervention is appreciated. I have some final suggestions to consider.

### Reviewer 1 – Comment 1

Medior suggestion:
- If possible, it would be interesting to see how many patients actually reached their goal, based on the accelerometer data. Do you have any insight in that? For example, in case you find that majority of patients in level 8 do not reach the goal of 30 minutes, that might help explain the negative outcome. And/or when you do not see any difference between I and C, than you know that the intervention did not promote mobilization and therefore did not improve physical functioning.

### *Reviewer 1 – Response 1*

*We only recorded the first mobility goal but did not record the mobility goals over time, mainly for two reasons. First, we aimed to keep the system as simple as possible to allow all stakeholders, including nurses or visitors, to change the mobility goals at any time and we did not use electronic boards because we used existing patient communication boards. Second, we refrained from regular, e.g. daily, registration of mobility levels to avoid that study team members interfere more frequently with patients in the intervention group compared to those in the control group. We are thus unable to give insights to this question, but acknowledged that the time mobilised did not differ between the groups as follows.*

> *"Until both, day 5 and discharge, the mobilisation time measured by accelerometers was similar in the control group and the intervention group."* (Results, p 13, lines 5–6)

> *"Secondary outcomes – such as mobilisation time, length of hospital stay, discharge destination, or 3-month survival – were not significantly influenced by the GDM program."* (Discussion, p 14, lines 23–24)
>
> *"Reaching a certain level of mobility may also have triggered discharge from hospital and biased mobilisation time, which did not differ between groups."* (Discussion, p 16, lines 10–11)

### Reviewer 1 – Comment 2

Minor suggestions:
- Did patient know what the GENEActiv measured? Is it thinkable that just knowing that their physical activity is measured might have resulted in an increase in PA in both groups? This might be a suitable topic for the discussion section.

### *Reviewer 1 – Response 2*

*All patients were informed that the GENEActiv measured physical activity. This has indeed been show to increase physical activity. (Clemes and Parker 2009; Konig et al. 2022) We added the following to the discussion:*

> *"Some study procedures such as measuring time mobilised with a wrist-worn device may have influenced mobility in both arms and thereby reduced differences between groups. (Clemes and Parker 2009; Konig et al. 2022)"* (p 16)

### Reviewer 1 – Comment 3

- As GENEActiv is not part of the intervention, so I would suggest to put the description somewhere else in the manuscript (eg. outcome measurement paragraph)

### *Reviewer 1 – Response 3*

*Thank you for this suggestion which we have implemented in the manuscript. (p 8, lines 8–16)*

### Reviewer 1 – Comment 4

- There is a discrepancy of sample sizes in the manuscript and the response to the reviewers. In the latter the authors state 'We expected to include 128 participants in the control group and 128 participants in the intervention group' . That does not seem to match with the sample size decription.

### *Reviewer 1 – Response 4*

*The numbers indicated in the previous response to the reviewers ("128 participants in the control group and 128 participants in the intervention group" are not correct. As correctly stated in the manuscript, we expected to include 128 participants in total, 64 participant in*

*each group. Additionally we accounted for a dropout of 20%, increasing the sample size to a total of 160 participants (80 per group). (p 9, lines 5–9)*

**Reviewer 1 – Comment 5**

- I find level 8 confusing. Walking 30 min or more at a speed of 2.0 km/h means a distance of 1000 meter. That is a big difference with 'walk more than 75 meter' . And also walk a stairs is completely different than walking 30 minutes. Furthermore the difference with level 7 is also confusing as this level is also 'walk more than 75 meter'. There is nothing that you can change about this now, but my experience is that very clear goal setting increases adherence. So this may have hampered adherence, and therefore outcomes.

7 Walk ≥ 75m 29 (35.8%)
8 Walk ≥ 75m (30 min or stairs) 0 (0%)

***Reviewer 1 – Response 5***

*The aim in level 8 may not have been clear enough, as the reviewer suggests. Given the high proportion of patients in level 7, in future studies, we would probably put more focus on defining the higher mobility goals. Nevertheless, patients in level 8 are not typically seen on our wards because they are usually discharged to other institutions or outpatient care, as stated in the Discussion. We addressed the reviewer's concern in the Discussion.*

> *"Level 8 is in line with studies using goals of 5,000 steps or physical activity for more than 30min, but may not have been clear enough. In our study, more than a third of participants were initially assigned to level 7, but none to level 8. In our experience, patients on level 8 are often quickly discharged, possibly also reflected in the short length of stay of around 5 days. (van Grootel et al. 2023; Gallardo-Gomez et al. 2023)"* (p 16, lines 4–8)

**Reviewer 1 – Comment 6**

- First sentence of clinical implications paragraph in discussion section you replaced physical activity with mobilization time. Physical functioning is your primary outcome measure so activity should probably be replaced with functioning.

***Reviewer 1 – Response 6***

*We added the primary outcome to this context. It reads now as follows:*

> *"In our trial, GDM, which fostered mobility without requiring additional resources that might not be available in everyday clinical practice, did not improve physical functioning or mobilisation time." (p 17, lines 24–25)*

**Reviewer 1 – Comment 7**

- In line with previous comment: In that same paragraph you say ‘To conclude, improving mobility…’ while the DEMMI (ie physical functioning) was your primary outcome. So again I think this should be replaced by physical functioning, or extended to ‘physical functioning and mobility’ . Please check the correct use of these terms throughout the manuscript.

### *Reviewer 1 – Response 7*

*We agree that we should stick to the primary outcome of the study, which was physical functioning, and added this to the first sentence of the paragraph.*

> *“To conclude, improving physical functioning and mobility during an acute medical hospitalisation with a pre-existing high level of care and short lengths of stay remains challenging.” (p 18, lines 3–4)*

**REFERENCES**


Clemes, S. A., and R. A. Parker. 2009. 'Increasing our understanding of reactivity to pedometers in adults', *Med Sci Sports Exerc*, 41: 674-80.

Gallardo-Gomez, D., J. Del Pozo-Cruz, H. Pedder, R. M. Alfonso-Rosa, F. Alvarez-Barbosa, M. Noetel, U. Jasper, S. Chastin, J. Ramos-Munell, and B. Del Pozo Cruz. 2023. 'Optimal dose and type of physical activity to improve functional capacity and minimise adverse events in acutely hospitalised older adults: a systematic review with dose-response network meta-analysis of randomised controlled trials', *Br J Sports Med*, 57: 1272-78.

Konig, L. M., A. Allmeta, N. Christlein, M. Van Emmenis, and S. Sutton. 2022. 'A systematic review and meta-analysis of studies of reactivity to digital in-the-moment measurement of health behaviour', *Health Psychol Rev*, 16: 551-75.

van Grootel, J., P. Bor, J. A. Netjes, C. Veenhof, and K. Valkenet. 2023. 'Improving physical activity in hospitalized patients: The preliminary effectiveness of a goal-directed movement intervention', *Clin Rehabil*, 37: 1501-09.
